# Dataset on the relationship between students’ attitude towards, and performance in mathematics word problems, mediated by active learning heuristic problem-solving approach

**DOI:** 10.1016/j.dib.2023.109055

**Published:** 2023-03-14

**Authors:** Robert Wakhata, Védaste Mutarutinya, Sudi Balimuttajjo

**Affiliations:** a University of Rwanda, College of Education (UR-CE), P.o Box 55 Rwamagana, Rwanda; b Mbarara University of Science and Technology; P.o Box 1410 Mbarara, Uganda

**Keywords:** Active learning, Attitude, Heuristic problem-solving, Mathematics, Secondary schools, Linear programming

## Abstract

The present data was applied to investigate the relationship between students’ attitude towards, and performance in mathematics word problems (MWTs), mediated by the active learning heuristic problem-solving (ALHPS) approach. Specifically, the data reports on the correlation between students' performance and their attitude towards linear programming (LP) word tasks (ATLPWTs). Four types of data were collected from 608 grade 11 students who were selected from eight secondary schools (both public and private). The participants were from two districts: Mukono and Mbale in Central Uganda and Eastern Uganda respectively. A mixed methods approach with a quasi-experimental non-equivalent group design was adopted. The data collection tools included standardized LP achievement tests (LPATs) for pre-test and post-test, the attitude towards mathematics inventory–short form (ATMI-SF), a standardized active learning heuristic problem-solving tool, and an observation scale. The data were collected from October 2020 to February 2021. All the four tools were validated by mathematics experts, pilot-tested, and found reliable and suitable for measuring students’ performance and attitude towards LP word tasks. To achieve the purpose of the study, eight intact classes from the sampled schools were selected using the cluster random sampling method. Of these, and by a toss of a coin, four were randomly assigned to the comparison group while the other four were randomly assigned to the treatment group. All teachers from the treatment group were trained on the application of the ALHPS approach before intervention. The participants’ demographic data (identification numbers, age, gender, school status, and school location) were presented together with the pre-test and the post-test raw scores obtained before and after intervention respectively. The LPMWPs test items were administered to the students to explore and assess their problem-solving (PS), graphing (G), and Newman error analysis strategies. Students' percentage scores in the pre-test and post-test were based on mathematizing word problems to optimization of LP problems. Data were analyzed in line with the purpose of the study, and the stated objectives. This data supplements other data sets and empirical findings on the mathematization of mathematics word problems, problem-solving strategies, graphing and error analysis prompts. This data may serve and provide some insights into the extent to which ALHPS strategies support students' conceptual understanding, procedural fluency, and reasoning among learners in secondary schools and beyond. The LPMWPs test items in the supplementary data files can also act as a basis for the application of mathematics in real-life scenarios beyond the compulsory level. The data is intended to develop, support, and strengthen students' problem-solving and critical thinking skills with the main goal of enhancing instruction and assessment in secondary schools and beyond.

**Specifications Table**

**Table:** 

Subject	Mathematics Education
Specific subject area	Mathematical Reasoning
Type of data	Table
How the data were acquired	The adapted data collection tools included: LP achievement tests (pre-test.pdf and post-test.pdf) [1,2,3,4,5] (content knowledge of the 11th-grade students in LP.pdf, sample LP word Problems.pdf) as model eliciting tasks [6,7,8,9,10,11,12,13], the ATMI-SF (ATMI-SF.pdf) [14,15,16,17,18,19,20,21,22,23,24,25], the ALHPS tool (ALHPSA.pdf) [26,27,28,29], an observation scale (classroom observation rubric.pdf) [30], the standardized focus group prompts (focus group prompts.pdf) and an interview guide (interview guide.pdf). All tools were validated by mathematics experts, pilot-tested, and found reliable and suitable as recommended by [31]. Data are available via: Mendeley Data, V2, doi:10.17632/cb4pmz9rnk.2
Data Format	Raw Filtered
Description of Data Collection	Data were collected from 608 11th-grade students. Students in the comparison group learned LP conventionally whereas students from the treatment group were taught using ALHPS strategies. The data were collected between October
	2020 and February 2021. The pretest was administered to both groups followed by an intervention and finally, a post-test. Descriptive and inferential data analysis was performed using SPSS (v.26) with Hayes PROCESS macro (v.4) [32].
Data source location	Town/Region: Mukono district, eastern Uganda, and Mbale district, eastern Uganda: Uganda In summary, the study was undertaken from a sample of 8 Secondary schools, 608 students, and 12 teachers of mathematics. Institution: 8 Secondary schools, 608 students, 12 teachers of mathematics. City/Town/Region: Mukono district, central Uganda, and Mbale district, eastern Uganda Country: Uganda
Data accessibility	The data is freely available and accessible to explore and reuse. Repository name: Mendeley. Data identification numbers: Mendeley Data, V2, doi:10.17632/cb4pmz9rnk.2 Direct URL to data: https://data.mendeley.com/datasets/cb4pmz9rnk/2
Related Research Articles	Wakhata Robert, Védaste Mutarutinya, and Sudi Balimuttajjo. 2022. "Effect of Active Learning through the Heuristic Problem-Solving Approach on Students' Achievement in Linear Programming." The International Journal of Science, Mathematics, and Technology Learning 29 (2): 67-85. doi:10.18848/2327-7971/CGP/v29i02/67-85. Wakhata Robert, Védaste Mutarutinya, and Sudi Balimuttajjo. 2022. “Pedagogical Content Knowledge of Mathematics Teachers: A Focus on Identifying and Correcting Sources of Students’ Misconceptions in Linear Programming.” The International Journal of Pedagogy and Curriculum, 29 (2): 23-45. Wakhata Robert, Védaste Mutarutinya, and Sudi Balimuttajjo. 2022. “Secondary school students’ attitude towards mathematics word problems.” Humanities and Social Sciences Communications, (2022) 9:444. 10.1057/s41599-022-01449-1.

## Value of the Data

These data are useful in the following ways:

• The data provides some insights into the application of suitable learning approaches, methods, strategies, and materials to support students’ learning, attitude, assessment, and evaluation for deeper and broader conceptualization and application of mathematics in real-world scenarios and vice versa.
• The data are also useful to policy-makers and curriculum designers in reviewing learning materials, hence, cultivating students’ critical thinking skills, performance, and attitude towards sustaining active learning principles and problem-solving strategies. This is vital for identifying and determining the professional development needs of subject teachers.
• The dataset is likely to build students’ confidence in learning mathematics. This may help future researchers in identifying the underlying gaps and possible remedies for learning the challenging mathematics word tasks (MWTs), and LP in particular. Students may make connections by linking prior knowledge and understanding to the new concepts.
• The present dataset provides a feasible basis for developing subsequent conceptual and theoretical frameworks since the research process is cyclic. Data may be used to replicate studies in mathematics education research and practice in different contexts and settings.

## 1 Objective

In this article, the dataset was used to investigate the relationship between students’ attitude towards, and performance in MWTs, mediated by the active learning heuristic problem-solving (ALHPS) approach. Descriptive and inferential statistics were used to analyze the data.

## 2 Data Description

This study adopted a mixed methods approach with a quasi-experimental (non-randomized) pre-test, post-test non-equivalent control group design [33,34]. Data were collected using four instruments; the LP achievement tests (LPATs), the attitude towards mathematics inventory short form (ATMI-SF), the active learning heuristic problem solving (ALHPS) strategies, and the observation tool. Research [35] shows that the challenges experienced in mathematics education are a by-product of those in education in general, and mainly span from policy, curriculum, instruction, learning, and information technology to infrastructure. To understand the challenges affecting students in LP, a total of 9 test items under 3 questions each for pre-test (LIPAT Pre.pdf) and post-test (TLIPAT Post.pdf) were validated by experts in mathematics education, pilot tested and found reliable to measure both students' attitude towards, and performance in LPMWPs. All the items were categorized dimensionally including and not limited to mathematization, graphical representation, and optimization. The dimensions and the validation process to support the curriculum materials at the 11th-grade level are provided in supplementary materials. All the LP test items were aligned according to each of the above three dimensions. The Dataset.sav (Dataset_Performance and Attitude.xlsx) is available, and it includes students' scores for the pretest and posttest. Students' demographic variables (Table 1) include identification numbers (individual students’ serial numbers), age (in complete years), gender (female or male), school location (urban or rural), school type (day or boarding), and school status (public or private). Students’ post-test scores and post-attitude were obtained after the pre-test to examine whether or not there exists a significant positive change in students' grades and attitude towards LPMWPs. Despite the usefulness of the data as outlined above, it is limited to measuring students’ active learning and problem-solving strategies in mathematics education. Thus, caution must be exercised when adopting these tools.

**Table 1:** Summarizes variable description the respective codes.

Variable	Codes
Experimental vs Comparison	1 = Experimental; 2 = Comparison
Gender	1 = Male; 2 = Female
Group	0 = Comparison; 1 = Experimental
School location	0 = Central; 1 = Western
ALHPS strategies	Graphing (G); Problem-solving (PS); Newman Error Analysis (NEA)
Att	Students’ attitude
AL	Active Learning
Age	Students’ age
School status	0 = Boarding; 1 = Day
School ownership	0 = Government; 1 = Private
Pre_test	Student's pre-test percentage scores
Post_test	Student's post-test percentage scores

## 3 Experimental Design, Materials and Methods

### 3.1 Formulation and development of research tools

The ALHPS strategies, the ATMI-SF, and the LPMWTs items were formulated with supporting literature from previous empirical findings, and following the Ugandan mathematics curriculum materials, designed and approved by the Ugandan National Curriculum Development Centre (NCDC). The LPMWTs exhibit model eliciting tasks, and demonstrate students' application of mathematics in real-life situations beyond the compulsory level at the 11th grade and vice versa. All the research tools were validated (content, construct, criterion and face) by three mathematics experts. The experts were selected based on their vast experiences in teaching and examining mathematics at the 11th-grade level and beyond. The process of validity and reliability were strictly adhered to before administering all the questionnaire items and related research tools to all the participants. A description of the validity and reliability procedure was provided to experts. All test items were reviewed and revised based on the feedback and suggestions from experts. Final copies were compiled for data collection. Finally, suitable validated and standardized tools were applied to collect data (see supplementary data files provided).

The data in the dataset were collected from 608 11th-grade students from central Uganda and eastern Uganda. Overall, 317 (52.1%) of the participants were randomly assigned to the comparison group while 291 (47.9%) were from the experimental group. The participants' ages ranged from 17 to 22 (*M*= 18.37, *SD* = .98) years. Of the 608 students, 267 (43.9%) students were male while 341 (56.1%) students were female. A multi-stage cluster random sampling method was adopted to select participants. Overall, eight intact classes were used as clusters. All schools were clustered according to performance (low, moderate, and high) irrespective of the status, location or type. Four schools were randomly selected by tossing a coin. Of these, two schools from eastern Uganda and two from central Uganda were randomly assigned to the experimental group. The remaining four schools from the aforementioned two regions were assigned to the comparison group. All the 11th-grade students for the academic year 2020/2021 constituted the sampling frame and units of analysis. All participants sat for a pre-test to examine the equivalence of the comparison and experimental groups in terms of the aforementioned baseline variables and characteristics. According to the constructivists [36,37], the pretest was administered to examine students' pre-existing knowledge and skills on LPMWTs. Of the 608 respondents, it was assumed that all the participants were at the same academic level at the time of administering the pre-test. Prior to the analysis, data were cleaned, coded (where applicable), entered in SPSS, and lastly screened for errors of omission and commission. Preliminary analysis were performed to examine whether or not data were symmetrical or asymmetrical based on the qualitative and quantitative assumptions. Finally, data were subjected to further statistical treatment (parametric or non-parametric tests). Both descriptive and inferential statistics were applied to analyze students' performance and attitude before and after an intervention.

### 3.2 Learning in the experimental group

The study involved a pre-intervention-intervention-post-intervention. First, pre-intervention was carried out in all eight schools to assess the baseline characteristic of participants. Second, the intervention phase was applied to examine the effect of the active learning heuristic problem-solving approach on students' performance and attitude towards LPMWPs. After the pretest had been administered to both groups (treatment and comparison), the teachers were trained to implement the learning of LPMWPs by applying the active learning heuristic problem-solving approach to the treatment group (mainly problem-solving, graphing, and error analysis prompts), while the comparison group learned LPMWPs conventionally (usual and mainly teacher-centered learning). Conventional educators and learners were taught through a traditional learning approach. Here, learning was centered on talk and chalk on the chalkboards for teachers; pen and paper for students. The teachers did not apply alternative learning methods such as demonstration problem-solving, inquiry approach and/or exploration, etc. The teachers neither applied whole-class students' involvement with varied examples nor student-centered learning and assessment. Several studies [e.g.,^38^,^39^,^40^] have shown that non-routine problems solved by students in typical active learning classrooms perform better than those taught conventionally. After applying the active learning heuristic problem-solving for the treatment group in learning LPMWPs for six weeks, a similar self-developed standardized LPMWPs post-test was administered to both groups. Students’ pen and paper responses were scored and analyzed by experts and both scores (pretest and posttest) were recorded as percentage scores (Table 2). We also ensured that students’ attitudinal change is examined before and after an intervention (Table 3). The attitude towards mathematics inventory-short form (ATMI-SF) was adopted. Important to note is that before administration, both tools were validated by experts, pilot-tested, and found to be reliable, suitable, and acceptable. The data set is freely available and may be accessed.

**Table 2:** Shows paired samples t-test.

					95% CI of Mean				
		Mean	S.D	S.E Mean	Lower	Upper	t df Sig. (2 tailed)		
	Student's posttest-pretest	11.90	15.94	.65	10.63	13.17	18.41 607 .000	607	.000

**Table 3:** Shows model for active learning and attitude.

	Coeff	SE	t	p	LLCI	ULCI
Constant	43.9672	4.1202	10.6712	0000	52.0588	-35.8757
Attitude	17.0392	1.1571	14.7263	0000	14.7669	19.3116
Active Learning	16.2792	1.1384	14.3005	0000	14.0436	18.5148

## Ethics Statements

Ethical clearance and approval were sought and granted by the Directorate of Research, Innovations and Ethics committee of the University of Rwanda, the corresponding authors' university. Thus, all procedures involving human participants were respected and streamlined following the ethical standards of the University's research and ethics committee. First, the research proposal was defended. Suggestions and corrections were integrated before the final proposal was submitted to the directorate of the research and innovation unit University of Rwanda College of Education (URCE) for ethical clearance. Finally, ethical clearance was granted under reference number 03/DRI-CE/061(b)/EN/gi/2020 (Research recomendation.pdf). Subsequent ethical clearance and permission were sought and granted by the ministry of education and sports (permission_ministry of education and sports.pdf) to conduct the study. Before accessing secondary schools, permission was also granted from the district education officers (permission_ district education officers.pdf) of the respective districts, and finally, from the headteachers of sampled secondary schools before accessing research participants. All respondents provided written consent (Consent form_students.pdf) with the assurance of confidentially, and anonymity and that data were for academic purposes only.

## CRediT authorship contribution statement

**Robert Wakhata:** Conceptualization, Methodology, Software, Data curation, Visualization, Investigation, Writing – original draft. **Védaste Mutarutinya:** Supervision, Validation, Writing – review & editing. **Sudi Balimuttajjo:** Supervision, Validation, Writing – review & editing.

## Declaration of Competing Interest

The authors declare that they have no known competing financial interests or personal relationships that could have appeared to influence the work reported in this paper.

## Data Availability

Data on the Effect of Active Learning Heuristic Problem-Solving Approach on Students’ Performance and Attitude towards Mathematics Word Problems (Original data) (Mendeley Data).

## Appendix Supplementary Materials

Image, application 1
