# Supplementary material for: Dataset on the relationship between students’ attitude towards, and performance in mathematics word problems, mediated by active learning heuristic problem-solving approach
Source: Data Brief. 2023 Mar 14;48:109055. doi: 10.1016/j.dib.2023.109055 (PMC10051018; doi:10.1016/j.dib.2023.109055)
Supplement: Supplementary file 1 [file mmc1.zip › Supplementary material for DIB/ALHPSA.pdf]

Questionnaire for Students on Application of Active Learning Heuristic Problem-Solving

No.	Item	SD	D	N	A	SA
1.	Teachers effectively applied GeoGebra software to demonstrate the relationship between equations and inequalities during the learning of linear programming.					
2.	Teachers effectively applied graph papers during the learning of linear programming.					
3.	Teachers effectively applied grid boards during the learning of linear programming to help us understand all concepts.					
4.	I am able to comprehend the given linear programming word task.					
5.	I am able to devise a plan before answering any linear programming task.					
6.	I am able to carry out a plan before finding the solution to any linear programming word task.					
7.	I am able to look back and verify procedures to my solutions after answering tasks on linear programming word problems.					
8.	I am able to adequately read linear programming word tasks.					
9.	I am able to understand all procedures for finding the solution of linear programming word problems.					
10.	I am always able to transform linear programming word problems into models (equations and inequalities).					
11.	I am able to process and adequately apply necessary skills needed for finding solutions to linear programming word problems.					
12.	I am able to encode (understand and convert) concepts for finding the solution of linear programming word problems.					
