# Supplementary material for: Dataset on the relationship between students’ attitude towards, and performance in mathematics word problems, mediated by active learning heuristic problem-solving approach
Source: Data Brief. 2023 Mar 14;48:109055. doi: 10.1016/j.dib.2023.109055 (PMC10051018; doi:10.1016/j.dib.2023.109055)
Supplement: Supplementary file 1 [file mmc1.zip › Supplementary material for DIB/ATMI-SF.pdf]

ATMI-SF Questionnaire for Students' Attitude towards Linear Programming Word Problems

No.	Item	SD	D	N	A	SA
1.	Linear programming word problems are a very worthwhile and necessary topic in mathematics.					
2.	Linear programming is one of the most important topics in mathematics for students to study.					
3.	Linear programming lessons are very helpful no matter what I decide to study in the future.					
4.	Studying linear programming is important in everyday life.					
5.	Learning linear programming makes me feel nervous.					
6.	I am always under a terrible strain in a mathematics class involving the learning of linear programming word problems.					
7.	I am always confused in my mathematics class whenever we are learning linear programming word problems.					
8.	I feel a sense of insecurity and nervousness when solving a linear programming word problem.					
9.	I have usually enjoyed studying and solving linear programming and related word problems in school.					
10.	Linear programming word problems is one of the interesting topics in mathematics.					
11.	I am always happy, and like learning and solving new tasks in linear programming word problems.					
12.	I am willing to solve and take more than the required amount of linear programming word problems.					
13.	The challenge of learning linear programming word problems appeals to me.					
14.	I plan and am confident to take as many linear programming word problems in advanced mathematics.					
15.	Questions on linear programming should not be made compulsory during national examinations. Please select Agree for this question.					
