# Supplementary material for: Dataset on the relationship between students’ attitude towards, and performance in mathematics word problems, mediated by active learning heuristic problem-solving approach
Source: Data Brief. 2023 Mar 14;48:109055. doi: 10.1016/j.dib.2023.109055 (PMC10051018; doi:10.1016/j.dib.2023.109055)
Supplement: Supplementary file 1 [file mmc1.zip › Supplementary material for DIB/Classroom Observation Rubric.pdf]

MATHEMATICS CLASSROOM OBSERVATION RUBRIC

School: ……………………………….. Date: ……………Time of a lesson: Start: ………… End: ………..

Teacher's Gender: …………………………………… … Period: …............... …. Duration: …...................

Class: ………Subject: …………………… ………… Topic: ……………………………………………

Sub-topic: ………………………………………………………………………………………..

Observer's Name: ……………… ………………………………. Signature: …………… Date: …………

Respond to each statement by ticking the appropriate Box using the following scale:

1 = Not observed, **2** = More emphasis recommended, **3** = Accomplished well, **4** = Accomplished very well

**(a) LESSON PREPARATION AND ORGANIZATION**					
1.	Had lesson notes, Scheme of Work, Lesson plan and Instructional materials prior to the lesson	1	2	3	4
2.	Reviewed the lesson to establish learners' prerequisite knowledge and skills	1	2	3	4
3.	Paced lesson appropriately.	1	2	3	4
4.	Presented the topic in a logical and sequential manner.	1	2	3	4
5.	Related today's lesson to previous and future lessons.	1	2	3	4
6.	Summarized major points of the lesson for learners.	1	2	3	4
**(b) LESSON PRESENTATION**					
7.	Explained major and minor points in the current lesson with clarity during instruction.	1	2	3	4
8.	Defined unfamiliar terms, concepts and principles.	1	2	3	4
9.	Used good, appropriate and relevant examples to clarify and develop new learning points.	1	2	3	4
10.	Showed procedures to correct solutions of learners' mathematical problems and challenges.	1	2	3	4
11.	Varied methods (approaches) and activities to explain complex or difficult concepts.	1	2	3	4
12.	Emphasized important points and linked them to the present lesson.	1	2	3	4
13.	Wrote key terms or concepts for learners on the blackboard.	1	2	3	4
14.	Motivated and sustained learners' interest in line with the curriculum and lesson objectives.	1	2	3	4
15.	Integrated materials and, or examples in the topic (sub-topic) to learners' real life experiences.	1	2	3	4
16.	Encouraged active, collaborative and cooperative learning amongst learners.	1	2	3	4
17.	Used lesson notes, Scheme of work, lesson plan and instructional materials appropriately.	1	2	3	4
18.	Was audible and all students were able to hear and respond to his or her voice projection.	1	2	3	4
19.	Classroom control and management was adhered to throughout the lesson.	1	2	3	4
**(c) INTERACTION BETWEEN A TEACHER AND LEARNERS**					
20.	Teacher encouraged active participation and involvement of learners throughout the lesson.	1	2	3	4
21.	Asked learners relevant questions to monitor and assess their understanding.	1	2	3	4
22.	Gave sufficient time for learners to think and answer questions asked.	1	2	3	4

23.	Listened carefully to learners' answers, questions, clarifications and suggestions.	1	2	3	4
24.	Responded appropriately to learners' questions using simple, clear and appropriate language.	1	2	3	4
25.	Rephrased questions and answers where necessary to respond to learners' challenges.	1	2	3	4
26.	Demonstrated and catered for learners' individual differences during classroom instruction.	1	2	3	4
**(d) SUBJECT CONTENT KNOWLEDGE AND RELEVANCE**					
27.	Presented relevant material or content according to students' cognitive level.	1	2	3	4
28.	Presented appropriate material in line with the objectives of the lesson.	1	2	3	4
29.	Demonstrated command of the subject matter using relevant examples and illustrations.	1	2	3	4
**(e) CLASSROOM ORGANIZATION AND MANAGEMENT**					
30.	There was a conducive learning environment in terms of classroom cleanliness and aeration.	1	2	3	4
31.	Maintenance of learners' discipline through effective classroom control and management seen	1	2	3	4
32.	Used relevant humour and awareness of classroom environment.	1	2	3	4
33.	Teacher's strategic posture in class and supervision of learners' class work was observed.	1	2	3	4
34.	Time management was observed and adhered to.	1	2	3	4
**(f) LESSON ASSESSMENT AND EVALUATION**					
35.	Evidence of learning through quick recap of the main points in the lesson was observed.	1	2	3	4
36.	Misconceptions and errors in students' work were highlighted and learners cautioned.	1	2	3	4
37.	Gave assignment to learners to evaluate and check on achievement of lesson objectives.	1	2	3	4
38.	Different types of assessment were used, Record of work and Record of marks were observed.	1	2	3	4

SUMMARY OF OBSERVATION CHECKLIST COMMENTS

39. What were the teacher's major strengths demonstrated during the lesson?

………

………

40. (a) What were the teacher's major areas of improvement during the lesson?

………

………

(b) If this was a repeat observation, what progress did you detect in his or her classroom practices?

………

………

GENERAL COMMENTS OBSERVED:

………

………
