# Supplementary material for: Dataset on the relationship between students’ attitude towards, and performance in mathematics word problems, mediated by active learning heuristic problem-solving approach
Source: Data Brief. 2023 Mar 14;48:109055. doi: 10.1016/j.dib.2023.109055 (PMC10051018; doi:10.1016/j.dib.2023.109055)
Supplement: Supplementary file 1 [file mmc1.zip › Supplementary material for DIB/Content Knowledge of the Eleventh Grade Students in LP.pdf]

Eleventh Grade Students' Content Understanding and Key Mathematical Ideas for Learning the Graphical Method of Solving Linear Programming Problems

Content (with Related Examples) for Solving LP Tasks	Key Mathematical Ideas for Learning LP in Secondary Schools
1. Representation of inequalities on a number line and writing down the solution set.<br>e.g., Using a number line, find the integral values of *x* which satisfy the sets: $\{3x > 2x + 5\} \cap \{3x < 32 - x\}$<br><br>2. Solving linear inequalities algebraically (non-graphical).<br>e.g., Solve: $\frac{x-3}{4} - \frac{x-2}{3} < 1$	▪ Correct use of a number line and related symbols.<br>▪ Knowledge of equations, i.e., the gradient of a straight line, negative gradient, positive gradient, finding the equation of the straight line in the form $y = \pm mx \pm c$,<br>$y = \pm mx, y = \pm m, x = \pm m$, etc.<br>▪ Solving linear equations correctly including the use of lowest common multiples, simplifying fractions, etc.<br>▪ Correct use of set notations e.g., $\cup, \cap, \emptyset, \in$, etc.<br>▪ The distinction between solving equations and inequalities.<br>▪ Correct use of symbols in solving linear equations.
3. Solving quadratic inequalities algebraically (non-graphical).<br><br>e.g., Solve: $3x^2 + 7x - 20 < 0$	▪ Identification of correct factors (critical points).<br>▪ Distinguishing critical values from solutions to the quadratic inequality.<br>▪ Review of the methods of solving quadratic equations. Showing students differences between the following graphs $y = \pm ax^2, y = \pm x^2, y = \pm ax^2 \pm bx, y = \pm ax^2 \pm bx \pm c,$<br>$y = (x \pm p)^2, y = (x \pm p)^2 + k,$<br>$y = \pm a(x \pm p)(x \pm p), = \pm(x \pm p)(x \pm p)$ etc. substitute = with symbols like $>, <, \geq$ and $\leq$. Students' knowledge of $b^2 - 4ac = 0$, $b^2 - 4ac \leq 0$ and $b^2 - 4ac \geq 0$<br>▪ Consistent use of mathematical symbols<br>▪ The distinction between inequalities with equations.<br>▪ Review of representation of equations and inequalities on the coordinate axes.
4. Solving linear inequalities graphically<br>e.g., Show by shading unwanted regions the region satisfying the inequalities: $x + y \leq 3$, $y > x - 4$, and $y \geq -3x$	▪ Obtaining correct coordinates from the given equations.<br>▪ Distinguishing dotted from solid lines based on mathematical symbols $<, >, \leq, \geq$.<br>▪ Identification of the correct feasible region.<br>▪ Correct use of scales on the coordinate axes.<br>▪ Decision on whether to shade wanted/unwanted regions.<br>▪ Identification of correct coordinates for plotting and testing the objective function to find the feasible region.<br>▪ Plotting graphs of equations instead of inequalities.
5. Solving simultaneous inequalities graphically.<br>e.g., On the same coordinate axes, draw the curve y= $4x^2$ and the line y = 1. Show by shading unwanted regions, the region represented by: y > 1 and y <	▪ The distinction between graphs of curves and straight lines.<br>▪ Correct use of mathematical symbolism when representing the coordinate axes.<br>▪ Failure to obtain coordinates from equations.<br>▪ Obtaining correct coordinates from the feasible region.<br>▪ Obtaining correct integral values and convex points.

$4x^2$. Hence, state the integral coordinates of the points which lie in the region $\{y > 1 \cap y < 4x^2\}$	
6. The graphical solution of a LP problem. An example is:<br>A school has organized a geography study tour for 90 students. Two types of vehicles are needed; Taxis and Costa buses. The maximum capacity of the taxi is 15 passengers while that of the Costa bus is 30 passengers. The number of taxis will be greater than the number of Costa buses. The number of taxis will be less than five. The cost of hiring a taxi is Shs.60,000 while that of the Costa bus is Shs. 100,000. There is only Shs. 600,000 available.<br>(a) If *x* represents the number of taxis and *y* the number of Costa Buses, write inequalities for the given information.<br>(b) Represent the inequalities on the graph paper by shading the unwanted regions.<br>(c) Find from your graph the number of taxis and Costa Buses which are full that must be ordered so that all the students are transported?<br>(d) Find the minimum and maximum cost of transporting 90 students?	▪ Students' comprehension of mathematical word problems, translating and writing correct inequalities from mathematics word statements.<br>▪ Students' ability to link mathematical symbols, variables, constraints, operations, algebraic expressions into mathematical language.<br>▪ Correct use of scales when representing equations on the coordinate axes.<br>▪ Writing the correct models and the objective function for optimization.<br>▪ Students' ability in solving equations simultaneously by graphical means.<br>▪ Review of basic mathematical vocabulary (at least, at most, greater than, less than, minimize, maximize, etc.<br>▪ Emphasize finding the correct feasible region.<br>▪ Identification of correct coordinates for optimization.<br>▪ Decision on shading unwanted regions and leaving out wanted regions.<br>▪ Adequate knowledge of the graphical solution of linear and quadratic equations simultaneously.<br>▪ Plotting graphs of inequalities, not equations.<br>▪ Correct labeling and use of axes (x and y respectively).<br>▪ Identification of correct points of intersection, x-intercepts, and y-intercepts from the graph.<br>▪ Ability to obtain coordinates from equations, and correctly plotting them on the same coordinate axes.<br>▪ Identifying the correct feasible region, obtaining integral values, and optimizing the coordinates.<br>▪ Ability to write correct equations, inequalities from the given feasible region.<br>▪ Interpretation of optimization terms (maximum or minimum), this leads to correct or incorrect substitutions and numerical values.<br>▪ Ability to use symbols to represent numbers, not objects.
