# Supplementary material for: Dataset on the relationship between students’ attitude towards, and performance in mathematics word problems, mediated by active learning heuristic problem-solving approach
Source: Data Brief. 2023 Mar 14;48:109055. doi: 10.1016/j.dib.2023.109055 (PMC10051018; doi:10.1016/j.dib.2023.109055)
Supplement: Supplementary file 1 [file mmc1.zip › Supplementary material for DIB/DEO Mbale.pdf]

ROBERT WAKHATA (Reg. No. 219014678)
C/O-UNIVERSITY OF RWANDA
COLLEGE OF EDUCATION
ACEITLMS
P.O BOX 55,
RWAMAGANA.

15th January 2021

Identified Headteachers
No objection from this office.
Permission granted as such
Forwarded

DISTRICT EDUCATION OFFICER
P. O. Box 701
Mbale - Uganda
Date: 22/[illegible]/2021

The District Education Officer
Mbale District,
P.O Box ……,
Mbale.

Dear Sir/Madam;

Re: Permission to Conduct Research from Secondary Schools in Mbale District

I am writing to request your office as referenced.

The purpose of this letter is to seek permission and voluntary participation in my research project entitled "Effect of Active Learning through the Heuristic Method on Students' Achievement and Attitude towards Linear Programming in Ugandan Secondary Schools". This research will engage teachers of mathematics and form four students from sampled secondary schools between 15th October 2020 and 25th February, 2021. Learners will also write Error Diagnostic Tests in **Inequalities and Linear Programming;** called Linear Programming Achievement Tests (LIPATs) before and after their teachers have applied visual resources and problem-solving during classroom instruction.

All data collected from sampled secondary schools in your district will be for academic purposes only. Therefore, any school is free to withdraw from this exercise any time if deemed necessary. In the process,

1. the teaching and learning (school timetable) in sampled secondary schools will not be interrupted.
2. all participants will sign consent forms.
3. this research will not be conducted during examination periods.
4. research will be carried out on Monday-Friday from 08:00am–05:00pm excluding weekends.
5. names of sampled secondary schools and those of teachers/learners will remain anonymous. Instead, pseudo names shall be used in disseminating research findings.
6. the research will be limited to the sampled secondary schools in the district.
7. part of the research findings and recommendations will be sent to the sampled schools upon completion of this research survey in order for S.4 learners to benefit from this study.
8. teachers will be interviewed, observed during teaching, audio or video taped upon granting the principal researcher (research team) permission.
9. all information obtained from this study shall be stored and guarded with utmost confidentiality and will be used for writing a Ph.D. Thesis and journal publications only.

I will be very grateful for your positive and official consideration.

Yours sincerely,

ROBERT WAKHATA
Ph.D. Student (Mathematics Education)
rwakhata@gmail.com
+250 785 333 300 / +256 701 692 986

Scanned with CamScanner
