# Supplementary material for: Dataset on the relationship between students’ attitude towards, and performance in mathematics word problems, mediated by active learning heuristic problem-solving approach
Source: Data Brief. 2023 Mar 14;48:109055. doi: 10.1016/j.dib.2023.109055 (PMC10051018; doi:10.1016/j.dib.2023.109055)
Supplement: Supplementary file 1 [file mmc1.zip › Supplementary material for DIB/Effect of ALHPSA on Students' Performance.pdf]

VOLUME 29 ISSUE 2

Downloaded by Robert Wakhata on Thu Nov 24 2022 at 00:47:07 AM CST

The International Journal of
Science, Mathematics and Technology Learning

Effect of Active Learning through the Heuristic Problem-Solving Approach on Students' Achievement in Linear Programming

ROBERT WAKHATA, VÉDASTE MUTARUTINYA, AND SUDI BALIMUTTAJJO

COMMON GROUND

THELEARNER.COM

THE INTERNATIONAL JOURNAL OF SCIENCE, MATHEMATICS AND TECHNOLOGY LEARNING
https://thelearner.com
ISSN: 2327-7971 (Print)
ISSN: 2327-915X (Online)
https://doi.org/10.18848/2327-7971/CGP (Journal)

First published by Common Ground Research Networks in 2022
University of Illinois Research Park
60 Hazelwood Drive
Champaign, IL 61820 USA
Ph: +1-217-328-0405
https://cgnetworks.org

The International Journal of Science, Mathematics and Technology Learning is a peer-reviewed, scholarly journal.

COPYRIGHT
© 2022 (individual papers), the author(s)
© 2022 (selection and editorial matter),
Common Ground Research Networks

All rights reserved. Apart from fair dealing for the purposes of study, research, criticism, or review, as permitted under the applicable copyright legislation, no part of this work may be reproduced by any process without written permission from the publisher. For permissions and other inquiries, please contact cgscholar.com/cg_support.

Common Ground Research Networks, a member of Crossref

EDITOR
Bill Cope, University of Illinois at Urbana-Champaign, USA
Mary Kalantzis, University of Illinois at Urbana-Champaign, USA

MANAGING EDITOR
Kortney Sutherland, Common Ground Research Networks, USA

ADVISORY BOARD
The Advisory Board of The Learner Research Network recognizes the contribution of many in the evolution of the Research Network. The principal role of the Advisory Board has been, and is, to drive the overall intellectual direction of the Research Network. A full list of members can be found at https://thelearner.com/about/advisory-board.

PEER REVIEW
Articles published in *The International Journal of Science, Mathematics and Technology Learning* are peer reviewed using a two-way anonymous peer review model. Reviewers are active participants of The Learner Research Network or a thematically related Research Network. The publisher, editors, reviewers, and authors all agree upon the following standards of expected ethical behavior, which are based on the Committee on Publication Ethics (COPE) Core Practices. More information can be found at https://cgnetworks.org/journals/publication-ethics.

ARTICLE SUBMISSION
The International Journal of Science, Mathematics and Technology Learning publishes biannually (June, December). To find out more about the submission process, please visit https://thelearner.com/journals/call-for-papers.

ABSTRACTING AND INDEXING
For a full list of databases in which this journal is indexed, please visit https://thelearner.com/journals/collection.

RESEARCH NETWORK MEMBERSHIP
Authors in *The International Journal of Science, Mathematics and Technology Learning* are members of The Learner Research Network or a thematically related Research Network. Members receive access to journal content. To find out more, visit https://thelearner.com/about/become-a-member.

SUBSCRIPTIONS
The International Journal of Science, Mathematics and Technology Learning is available in electronic and print formats. Subscribe to gain access to content from the current year and the entire backlist. Contact us at cgscholar.com/cg_support.

ORDERING
Single articles and issues are available from the journal bookstore at https://cgscholar.com/bookstore.

OPEN RESEARCH
The International Journal of Science, Mathematics and Technology Learning is Hybrid Open Access, meaning authors can choose to make their articles open access. This allows their work to reach an even wider audience, broadening the dissemination of their research. To find out more, please visit https://cgnetworks.org/journals/open-research.

DISCLAIMER
The authors, editors, and publisher will not accept any legal responsibility for any errors or omissions that may have been made in this publication. The publisher makes no warranty, express or implied, with respect to the material contained herein.

Downloaded by Robert Wakhata on Thu Nov 24 2022 at 00:47:07 AM CST

Effect of Active Learning through the Heuristic Problem-Solving Approach on Students' Achievement in Linear Programming

Robert Wakhata,[1] University of Rwanda, Rwanda
Védaste Mutarutinya, University of Rwanda, Rwanda
Sudi Balimuttajjo, Mbarara University of Science and Technology, Uganda

Abstract: This study examined the effect of active learning through the heuristic problem-solving approach on students' achievement in linear programming (LP). The participants were 608 eleventh-grade students from eight public and private secondary schools in central and eastern Uganda. Multistage cluster sampling was used to select participants. A mixed-method approach was used. Specifically, a pretest-posttest non-equivalent quasi-experimental design was adopted. Classroom observation and LP diagnostic tests constituted data collection tools. Eight secondary schools participated; four applied the heuristic problem-solving approach while the remaining four applied the usual conventional instructional approach. To overcome the effect of non-randomization, data were matched using propensity score matching (PSM), and the Difference in Difference (DID) estimand was used for analysis. The findings revealed that the heuristic problem-solving approach had a significant impact on students' achievement in LP (DID = 15.40). Generally, although both groups (comparison vs treatment) appear to have improved their average scores (4% vs 20%), the paired sample t-test (t(607) = 18.41, p = .000), and the effect size (Cohen's d = 0.65) showed that students from the treatment group exhibited proficiency in solving LP problems compared to their counterparts from the comparison group. Students from central Uganda performed better. Generally, the entire classroom observation of the treatment group revealed that students' mathematization of LP problems, their conceptual and procedural knowledge, understanding, and application of requisite knowledge in solving novel non-routine LP and related problem contexts improved. The study recommends adopting the heuristic problem-solving approach in secondary school mathematics to enhance instruction and assessment.

Keywords: Equations, Heuristic Problem-Solving Approach, Inequalities, Linear Programming, Model Eliciting Activities

Introduction

Developing students' problem-solving abilities has been a major area of focus in the twenty first-century education systems. During the learning and assessment of mathematics, several ad hoc problem-solving methods are needed to find solutions to mathematics tasks. Different approaches have been designed to help teachers effectively develop students' mathematical knowledge and skills. At the same time, specific methods as well as assessment tools for learning have been designed to help students retain knowledge and skills, and apply them in solving new and more challenging problems in new contexts (Jonassen 2011; Kilpatrick, Swafford, and Findell 2001; NCTM 2014; Polya 2004; Stein et al. 2000). Teachers struggle to develop and foster students' cognitive, affective, and psychomotor knowledge domains through problem-solving so that they may enhance students' critical thinking, procedural fluency, relational and conceptual understanding, and application of the knowledge and skills learned in solving real-world problems (Angraini and Wahyuni 2020; Samuelsson 2010). Problem-solving is therefore an integral part of day-to-day living experiences since every aspect of life is about problem-solving (Jonassen 2011). To develop and promote learners' mathematical proficiency, the National Council of Teachers of Mathematics (NCTM 2014) recommends the adoption of five interrelated strands of "conceptual understanding, procedural fluency, strategic competence, adaptive reasoning, and productive disposition" (NCTM 2014, 7). It is from the above perspective

[1] Corresponding Author: Robert Wakhata, African Centre of Excellence for Innovative Teaching and Learning Mathematics and Science (ACEITLMS), University of Rwanda, College of Education. PO Box 55, Rwamagana, Rwanda, Kigali. email: rwakhata@gmail.com

The International Journal of Science, Mathematics and Technology Learning
Volume 29, Issue 2, 2022, https://thelearner.com
© Common Ground Research Networks, Robert Wakhata, Védaste Mutarutinya, Sudi Balimuttajjo, All Rights Reserved. Permissions: cgscholar.com/cg_support
ISSN: 2327-7971 (Print), ISSN: 2327-915X (Online)
https://doi.org/10.18848/2327-7971/CGP/v29i02/67-85 (Article)

Downloaded by Robert Wakhata on Thu Nov 24 2022 at 00:47:07 AM CST

that different school mathematics curricula worldwide support and emphasize students' acquisition of problem-solving skills (Shikuku and Amadalo 2015; Mushlihah and Sugeng 2018).

Linear programming (LP) is a branch of operations research and is one of the topics taught to eleventh-grade Ugandan lower secondary school students. Linear programming problems are formulated from decision-making word problems and are symbolically represented (Kenney et al. 2020). The solution to LP problems consists of decision variables that reflect the decisions to be made and an objective function to be optimized (minimized or maximized) subject to a set of mathematical constraints on the variables. In this article, the content is limited to an introduction to LP concepts and is aimed at developing a foundation for more complex LP problems later at the university and other tertiary institutions of learning. The unit or topic of LP is also referred to as linear mathematical optimization and is taught under quantitative techniques in tertiary institutions. In this research, the graphical solution of LP problems is mainly emphasized. According to the National Curriculum Development Centre (NCDC 2018), the topic of linear programming is introduced to secondary school students to develop their decision-making skills and problem-solving abilities. A typical LP problem in two dimensions is written in the form:

$$\begin{aligned} &\text{min./max.}\, x_1 \pm x_2 \\ s.t\ &ax_1 \pm bx_2 \leq\geq c \\ &dx_1 \pm ex_2 \leq\geq f \\ &gx_1 \pm hx_2 \leq\geq i \\ &x_1 \geq 0 \\ &x_2 \geq 0 \end{aligned}$$

To solve the above LP problem, the non-negative constraints ($x_1 \geq 0$ and $x_2 \geq 0$) and the set of points satisfying the main constraints ($ax_1 \pm bx_2 \leq \geq c$, $dx_1 \pm ex_2 \leq \geq f$ and $gx_1 \pm hx_2 \leq \geq i$) are plotted on the same coordinate axes. The feasible region is identified at the intersection of the lines (after shading unwanted regions). The corner points of the bounded half-plane constraint set are then substituted into min/max $x_1 \pm x_2$, which is called the objective function and used for finding optimal solutions. Question 1 below shows an example of a typical LP problem.

Question 1: A factory makes two kinds of bottle tops "Coca-Cola" tops and "Pepsi Cola" tops. The same equipment can be used to make either. In making Coca-Cola tops, one man can supervise ten machines and this batch will give a profit (amount) of pounds sterling (£) 50 per week. Pepsi Cola tops yield a profit of (£) 250 a week, using 25 machines and 8 men. There are 200 machines and 40 men available. By taking $\boldsymbol{x}$ batches of Coca-Cola tops and $\boldsymbol{y}$ batches of Pepsi Cola tops; write down inequalities for the:

a) (i) number for machines used (ii) number of men employed (iii) expression for profit, *P*.
b) Using inequalities in (a) above, draw a suitable graph on the same coordinate axes and clearly show the feasible region.
c) From your graph, determine the number of Coca-Cola tops and Pepsi Cola tops which should be made to obtain the maximum profit. Hence, find the maximum profit.

When trying to solve the above (and other related) LP optimization problem (Question 1), some students may struggle to understand and/or transform mathematics word problem statements into symbolic models in the form of equations and inequalities. Students further face challenges in determining and optimizing the feasible area and vice versa. Looking at the Ugandan mathematics curriculum materials, it was observed that the LP content in the Ugandan lower secondary school mathematics textbooks, other curriculum materials, and teachers' reference books foster the students' procedural understanding more than conceptual understanding. Yet, research shows that there exists a linkage between the concepts students learn and the approaches used by teachers to introduce the concepts (Kilpatrick, Swafford, and Findell 2001). Consequently, if relational

Downloaded by Robert Wakhata on Thu Nov 24 2022 at 00:47:07 AM CST

concepts are adequately introduced and mastered, students' conceptual understanding may be guaranteed. Students' challenges in learning and solving LP problems are perhaps a consequence of various difficulties encountered in learning algebra, equations, and inequalities. There seems to be a mismatch between the mathematics curriculum expectations and the actual learning outcomes.

In a related genre, the approaches employed by some mathematics teachers to introduce equations and inequality concepts and their connection to the students' conceptual and procedural understanding of LP may be inadequate. In introducing LP concepts (as non-routine tasks), teachers are expected to review students' knowledge of algebra and related symbolism, the use of the number line, equations, inequalities, set theory, coordinate geometry, and the correct application of basic mathematical rules, operations, and principles. Thus, this research sought to answer the research question of whether or not employing active learning through the heuristic problem-solving approach could deepen and broaden students' understanding of LP concepts. We hoped that the observed learning gaps and challenges in LP could provide opportunities for students to acquire basic mathematical concepts for higher education, and for teachers to develop suitable learning approaches.

Although empirical literature from different settings and contexts generally attribute students' difficulties in mathematics to the nature of the curriculum, teaching-learning environment, conventional learning approaches, and students' attitude toward mathematics (e.g., Alex and Mammen 2018; Allsopp, Kyger, and Lovin 2007; Budak 2015; De Jesus, Cyrino, and Oliveira 2015; Jonassen 2011; Mukuka, Balimuttajjo, and Mutarutinya 2020; NCTM 2014; Polya 2004; Quintero and Rosario 2016; Samuelsson 2010; Smith and Stein 1998; Stein, Grover, and Henningsen 1996), an extensive body of research shows that students' difficulties in LP generally stem from their inability in comprehending the basic knowledge of equations and inequalities (Almog and Ilany 2012; Bazzini and Tsamir 2004; Jupri and Drijvers 2016; Kenney et al. 2020; Makonye and Shingirayi 2014; Pongsakdi et al. 2020; Tsamir and Almog 2001; Tsamir and Bazzini 2004; Verikios and Farmaki 2010).

Kilpatrick, Swafford, and Findell (2001) have argued that different problems (ranging from simple to complex) require different problem-solving approaches. Moreover, for effective learning, proficiency in mathematics problem-solving depends not only on students' engagement, their cognition, and ability but also on the interaction with the curriculum (nature of the problems), the teachers' pedagogical content knowledge, and their professional development (Abdelsamad and Kandeel 2021; NCTM 2014; Stein et al. 2000). Besides, proficiency in problem-solving is partly dependent on students' levels of cognitive demand based on their stages of growth and development (Allsopp, Kyger, and Lovin 2007). Kilpatrick, Swafford, and Findell (2001) asserted and argued that students' mathematical proficiency is a gradual process and mainly depends on the quality of instruction and the types of tasks given to the students during instruction.

> Proficiency in mathematics is acquired over time when students have fully identified mathematical relationships between the previous and subsequent content. Each year they are in school, students ought to become increasingly proficient. For example, third graders should be more proficient with the addition of whole numbers than they were in the first grade. (Kilpatrick, Swafford, and Findell 2001, 135)

The Heuristic Problem-Solving Strategies (HPSS)

According to Polya (2004) in his book "How to Solve It," heuristic problem-solving entails "understanding the problem, devising a plan, carrying out a plan, and looking back" (Polya 2004, 201). Kilpatrick, Swafford, and Findell (2001) proposed a more comprehensive heuristic problem-solving approach based and in relation to Polya's stages of problem-solving. According to Kilpatrick, Swafford, and Findell (2001), proficiency in problem-solving entails five strands, which include conceptual understanding, procedural fluency, strategic competence, adaptive reasoning, and productive disposition. To Kilpatrick, Swafford, and Findell, the above strands are

Downloaded by Robert Wakhata on Thu Nov 24 2022 at 00:47:07 AM CST

intertwined and aid students' acquisition and development of cognitive, affective, and psychomotor domains of knowledge. The authors believed that

> as a child gains conceptual understanding, computational procedures are remembered better and used more flexibly to solve new problems. In turn, as a procedure becomes more automatic, the child is enabled to think about other aspects of a problem and to tackle new kinds of problems, which leads to new understanding. When using a procedure, a child may reflect on why the procedure works, which may, in turn, strengthen existing conceptual understanding. (Kilpatrick, Swafford, and Findell 2001, 134)

Several empirical studies have applied the heuristic problem-solving approach based on different theoretical perspectives in different contexts and settings. For instance, Ofori-Kusi and Mogari (2017) investigated the effect of the heuristic problem-solving approach on learners' conception and achievement in algebra. The findings revealed that the heuristic teaching approach improved students' average scores in algebra. The study further recommended the integration of this approach in learning other mathematical concepts and topics. Moreover, algebraic expressions, rules, and principles are directly linked to the mathematization of mathematics word problems. Whereas Ofori-Kusi and Mogari's study was conducted in the primary school context, we believe that secondary school students' proficiency in learning LP largely depends on a basic prior conceptual understanding of algebraic expressions and related concepts.

Despite the growing evidence of research into the relationship between several instructional methods, varied mathematics tasks, and students' performance (De Jesus, Cyrino, and Oliveira 2015; Stein et al. 2000; Suh 2007), there is still limited understanding of how students and teachers relate during designing specific tasks and linking the tasks to suitable approaches that may enhance students' performance, proficiency, critical thinking, conceptual understanding and retention of knowledge and skills. The integration of students' engagement to the HPSS is, therefore, inevitable in addressing specific mathematics learning deficiencies. The HPSS are a form of active learning instructional approach that develops and fosters a student's critical thinking and engagement in science, technology, engineering, and mathematics (STEM). If applied effectively, active learning through the heuristic problem-solving approach may enhance students' deeper and broader mathematical conceptual understanding. The HPSS are the theoretical and practical application of problem-solving techniques that provide approximate solutions to business, STEM, and societal problems that cannot be solved exactly. The HPSS have been applied in different contexts and settings and have yielded significant results (e.g., Abu Bakar and Ismail 2020; Apkarian et al. 2021; Feden and Vogel 2003; Ting, Lam, and Shroff 2019).

The Learning of Linear Programming in the Ugandan Context

To effectively teach mathematics, the Ugandan lower secondary school curriculum emphasizes problem-solving, which is embedded within the objectives of learning mathematics (NCDC 2008, 2018). Moreover, by the end of the eleventh grade (locally called senior four), students should be able to acquire basic, comprehensive, theoretical, and practical mathematical knowledge and skills for solving community problems. Indeed, students should demonstrate an understanding and be able to mathematize word problems and effectively apply the basic mathematical knowledge, principles, skills, and understanding of LP concepts in solving real-life, novel, non-routine world problems. The students' problem-solving abilities are inevitable for the growth and development of a holistic person as well as for national development (NCDC 2018). However, the Uganda National Examinations Board (UNEB) reports on the work of candidates at the Uganda Certificate of Education (UCE) reveal persistent high failure rates in mathematics, and in particular, LP, which seems challenging to most students (UNEB 2016, 2018, 2019, 2020). In this topic, majority of students have consistently failed to form correct inequalities from LP word problems (mathematize),

Downloaded by Robert Wakhata on Thu Nov 24 2022 at 00:47:07 AM CST

use the correct scale, correctly represent the formulated inequalities on the graph, correctly label the axes, list integral solutions, and optimize the feasible region. In this context, we believed that application of effective learning approaches that promote students' conceptual and procedural understanding may enhance students' abilities in mathematics, and LP in particular.

The Conceptual Framework and Literature Review

This research employed a problem-solving framework developed by Kilpatrick, Swafford, and Findell (2001). Kilpatrick, Swafford, and Findell developed five interwoven strands for achieving mathematical proficiency. The conceptual framework is based on the ideas of Kilpatrick et al. that proficiency in mathematics problem-solving entails students' holistic achievement of the three domains: cognitive, affective, and psychomotor. Thus, the methods of teaching mathematics should emphasize and promote learners' acquisition and application of the knowledge, beliefs, and skills in solving real-life problems in the communities they live. In this regard, Kilpatrick et al. summarized five interconnected strands which aid the acquisition of students' mathematical proficiency through problem-solving. The five strands are conceptual understanding, procedural fluency, strategic competence, adaptive reasoning, and productive disposition. The objective of the heuristic teaching approach was to develop and foster students' LP abilities in the above strands. In formulating the LP diagnostic tests for instruction and assessment, six principles (reality, model construction, self-assessment, construct documentation, construct shareability and reusability, and effective prototype) for creating modeling eliciting activities (MEAs) were applied (Lesh et al. 2000). In the context of LP, MEAs refer to specific open-ended tasks that were designed to engage and challenge students to reason and think critically in building models for solving real-world non-routine problems. This was justified by the fact that LP tasks are related to mathematical modeling. Thus, the tasks were all thought-revealing and modeling eliciting.

It is important to note that by examining answers to LP problems and related non-routine problems, the student's abilities and challenges in problem comprehension, interpretation, and mathematization were revealed explicitly. The process of finding the solution to the above LP problem and examining students' paper-and-pen responses revealed students' LP proficiency or their inability in problem-solving. The characteristics of the above LP problem provided in Question 1 can, therefore, be contextualized based on Kilpatrick et al.'s five strands.

Conceptual understanding is about the comprehension of LP mathematical word linguistic problems, requisite concepts of mathematical operations, rules and principles, set theory, number system, coordinate geometry, equations and inequalities, and their relationships. Conceptual understanding, therefore, entails coherency and the application of basic algebraic concepts of problem-solving. Thus, students recognize, plan, and summarize the information from a LP word problem by writing down the knowns and the unknowns and establishing if the given information is sufficient. This is the starting point and helps to highlight the connection between students' strengths and weaknesses in mathematizing the LP problem for effective learning. The student's prior knowledge and understanding is used to devise effective learning approaches.

Procedural fluency involves figuring out the connection between what is known and unknown. It takes into account the application of knowledge and skills of basic algebraic concepts to efficiently and effectively carry out mathematical computations following acceptable mathematical procedures and principles. In this regard, mathematics teachers design suitable instructional strategies, by designing similar and different challenging tasks for students. Consequently, students think of other auxiliary problems, find solutions to them, and draw relational meaning and connection in trying to solve subsequent problems. Students' proficiency in applying basic algebraic principles to perform mathematical computations without memorization and reproduction of basic mathematical principles in number theory, equations, and inequalities exhibits procedural fluency. Consequently, students conjecture, justify, and predict the solutions to the stated and related problems.

Downloaded by Robert Wakhata on Thu Nov 24 2022 at 00:47:07 AM CST

Strategic competence entails verification and proves that the procedures adopted or adapted are correct. This strand reveals students' ability or inability to use mathematical rules and principles, and conceptual understanding to formulate, represent, and effectively solve mathematical problems. In the context of LP, students are expected to correctly write inequalities from the given LP word problem, obtain coordinates from the formulated equations, and graph inequalities on the same coordinate axes. In this strand, students begin to appreciate the relationship between different tasks and their relational understanding during problem-solving. The teachers, therefore, plan effective instructional problem-solving approaches and strategies for appraising students' learning.

Adaptive reasoning implies students' ability to reason logically and sequentially while reflecting and explaining the formulated LP models. Whether the LP models are correct or wrong, the students should demonstrate their capacity and ability to represent, justify, and obtain meaning from coordinates and equations drawn on the same coordinate axes. Students' ability or their inability, through adaptive and relational reasoning, to formulate and critically evaluate the LP models is paramount. The application of mathematical rules and basic algebraic principles leads to the formulation of correct models. Students apply their reasoning and problem-solving abilities to ensure that the arguments to the solution sketches are sufficient.

Productive disposition is the final strand where the final solution is examined with the given problem. In other words, is the solution to the given LP problem meaningful? This can be established by looking back to the original question and verifying if the procedures and answers to the problem are relevant. In this context, the productive disposition is based on the notion that the optimal solutions to a particular LP problem are meaningful. To the teacher, the productive disposition is the function of the students' holistic understanding of the entire LP problem and how the knowledge and skills attained in one problem can be applied in subsequent problems. The strengths and weaknesses in learning LP can effectively be addressed by the teacher during planning, teaching, and assessment. To develop students' mathematical proficiency (LP in particular), the inadequacies in students' mathematical thinking should be treated as opportunities for subsequent learning. In this case, teachers develop suitable content and methods to justify or support mathematical arguments during the learning process. Students' learning challenges may also be addressed during the teachers' professional development courses, and feasible strategies to overcome specific learning challenges can be sought.

It is important to note that students' learning and understanding of mathematical concepts largely depend on the teachers' classroom instructional practices in consistently integrating the above Kilpatrick, Swafford, and Findell five strands. The main reason why students may not correctly mathematize several mathematics word problems is due to their failure to link school mathematics to everyday life experiences. In this research, students' intuitive solutions to the novel non-routine LP problems formed the basic components that were evaluated for mathematical proficiency. As earlier stated, and to provide answers to the effect of active learning through the heuristic problem-solving approach on students' achievement in LP, the following hypothesis was tested.

> H_{o1}: There is no statistically significant difference between the mean achievement scores of students taught using active learning through the heuristic problem-solving instructional approach and those taught using the usual conventional axiomatic method of learning linear programming.

Method

The Population, the Sample, and Sampling Techniques

All the eleventh-grade (senior four) students from public and private secondary schools in Eastern and Central Uganda for the school academic year 2020/2021 constituted the sampling frame. The sample consisted of intact eleventh-grade classes from eight secondary schools in Mukono district

Downloaded by Robert Wakhata on Thu Nov 24 2022 at 00:47:07 AM CST

of central Uganda and Mbale district of eastern Uganda. The multistage cluster sampling method was used to purposively choose four secondary schools from each region. First, the two regions were purposively selected based on students' previous academic performance at UCE. Second, four secondary schools were selected from each region (two schools from each region were assigned to the experimental group). According to Fraenkel, Wallen, and Hyun (2011), cluster sampling was suitable because it is cost-effective in terms of time and money.

Selected secondary schools for the experimental group were approximately 250 km away from one another for two reasons—first, to avoid diffusion and spurious results and second, to compare and contrast students' abilities and achievements in LP in the two regions. The units of analysis in their intact classes were all selected as clusters to represent the entire sampling frame. All the 11th grade students from the sampled schools participated in the study. There were 639 students at the time of administering the pretest. During data entry and analysis, the scores of thirty-one students from the experimental group were excluded due to missing posttest average scores. The students missed the posttest because they were absent on the day the posttest was administered. Consequently, the findings reported in this research were based on data from 608 students. All students assented and consented before they voluntarily participated. Of the 608 participants, 342 (56.25%) were female and 266 (43.75%) were males. The students' age ranged from 17 to 20 ($M = 17.70$; $SD = 0.95$).

Research Design

The study adopted a mixed-method approach to adequately address the stated hypothesis (Creswell and Plano 2018; Creswell 2014). This design was appropriate as it enabled the researcher to benefit from the advantages of triangulation by concurrently collecting both quantitative and qualitative data. A quasi-experimental non-equivalent control group design was applied. Specifically, the embedded exploratory sequential mixed methods research design was adopted. Phase 1 focused on a qualitative classroom observation to investigate the teaching approaches used by the teachers in all the eight secondary schools and assess the baseline characteristics of each school. The main objective was to use the preintervention classroom observation to control for some intervening variables that may directly or indirectly influence students' achievement in LP other than the heuristic problem-solving instructional approach. The qualitative data also revealed confounding variables that needed to be controlled. In Phase 2, a pretest was administered followed by treatment, and finally a posttest. Phase 3 included testing the hypothesis and the interpretation of results based on both qualitative and quantitative data. Using the stated approach and design helped to compare and contrast the students' average scores for the pretest and the posttest between the treatment group and the comparison group in the two regions of central Uganda and eastern Uganda respectively. Data were collected between October 2020 and February 2021.

Data Collection

The classroom observation scale, the pretest, and the posttest LP achievement tests were the three instruments used for data collection.

Classroom Observation

To compare and contrast the learning approaches in all the eight sampled schools, the classroom observation scale (Shafer, Wagner, and Davis 1997) was adopted. The tool was validated by six expert mathematics educators (two senior secondary teachers of mathematics, two teacher-trainers at National Teachers' Colleges, and two lecturers for mathematics education). The experts were selected based on their vast experience in learning and examining mathematics. The observation tool was found to be valid, relevant, and suitable. Before data collection, the tool was pilot tested, approved, and found reliable ($\alpha = 0.82$). Classroom observation in all eight secondary schools was done between October 2020 and February 2021.

Downloaded by Robert Wakhata on Thu Nov 24 2022 at 00:47:07 AM CST

Each intact class was observed three times by the principal researcher. Subsequent classroom observations were done by eight research assistants twice a month to consistently assess the nature of learning LP in the selected schools. The observed lessons and classroom activities were recorded using the validated monitoring tool. The pre-intervention observation of the entire learning process indicated that different baseline characteristics, for instance, location of the school (central or eastern), school status (day or boarding), school ownership (private or public), and students' previous academic abilities (low, average, and high) influenced the students' learning environment and achievement in LP.

The Pretest and the Posttest

The pretest and the posttest LP achievement tests were isomorphic equivalent. To achieve this, LP tests were rephrased to reflect the same Ugandan mathematics curriculum content. The main objective of the pretest was to find out students' prior conceptual knowledge, procedural knowledge, and skills of solving LP problems by graphical method. The posttest was rephrased to measure students' problem-solving abilities after the pretest. Intervention was based on the heuristic methods of solving LP tasks learned and practiced. Both the pretest and posttest items were based on the content from the Ugandan lower secondary school mathematics curriculum designed and approved by the NCDC. To maintain students' levels of cognitive demand, the content for LP from the mathematics teaching syllabus was merged with that of the UNEB syllabus. By combining the content in the two syllabi, it was anticipated that students' conceptual understanding of LP concepts, application of the acquired knowledge and skills to novel and non-routine real-life problems, and confidence in answering related questions would be enhanced.

Six principles (model construction principle, reality principle, self-assessment principle, construct documentation principle, construct shareability and reusability principle, and effective prototype principle) were followed to develop and formulate LP mathematics word achievement tests (Kilpatrick, Swafford, and Findell 2001; Lesh et al. 2000), as thought-revealing and model-eliciting activities. The same procedure was used for designing LP classroom learning activities to suit the heuristic problem-solving approach. The diagnostic test items for pretest and posttest were also validated by mathematics experts (two senior secondary teachers of mathematics, two teacher-trainers at National Teachers' Colleges, and two lecturers for mathematics education) before they were pilot tested outside the study sample ($n = 40$). Piloting the tests helped to make adjustments in questions to suit students' academic level, language structure, and duration. Validity (face and content) and reliability (test-retest = ICC = 0.83, 95% CI: 0.67–0.94) of the tests were pilot tested and the tests were found to be suitable, reliable, and acceptable. Juras (2016), Koo and Li (2016), and Oosterwijk, van der Ark, and Sijtsma (2019) recommend stating both the values of reliability and estimation precision. All the teachers assigned to the experimental group were trained on the appropriateness of implementing the heuristic problem-solving approach in their respective classrooms while those in the comparison group taught conventionally.

Administration of Pretest and Posttest

Of the 608 students, 285 from the experimental group and 317 from the comparison group sat LP achievement tests (pretest and posttest). Students spent approximately one hour completing LP tasks in each test. The two diagnostic tests were administered between October 2020 and February 2021. Students' participation in answering the tests was completely voluntary. A team of experienced mathematics teachers (as experts) scored the students' assessment scripts using a harmonized and uniform marking guide. To avoid bias during the marking exercise, no expert marked the entire students' script. However, a "conveyor belt system" adopted from UNEB where every expert marked one question only and passed the students' answer script to the next expert was adopted and applied during the entire marking exercise. Any inconsistencies that arose from

Downloaded by Robert Wakhata on Thu Nov 24 2022 at 00:47:07 AM CST

the marking exercise were addressed by a senior independent expert by sampling 10 percent of the students' marked scripts for moderation. Students' marks were converted to 100 percent and recorded in SPSS software for further statistical treatment. The posttest was aimed at assessing students' achievement in LP between comparison and experimental groups, after an intervention.

Data Analysis

The data recorded in SPSS (Version 26) was exported to STATA software (Version 13) to perform propensity score matching (PSM) analysis due to the researchers' limited analytical skills in using SPSS to compute PSM. The PSM method was applied to balance non-equivalent groups and to control for selection bias on a set of baseline characteristics (Rosenbaum and Rubin 2007). This was due to the non-random assignment of subjects to the treatment group. The purpose of PSM was to adjust for pre-treatment observable differences between treatment and comparison groups, mimic experimental designs, and subsequently estimate the treatment effect. Propensity score (PS) is the conditional probability of subjects being assigned to the treatment group as a function of baseline characteristics (Fan and Nowell 2015; Lane and SherRhonda 2015; Rosenbaum and Rubin 2007).

The common support across propensity score distributions was used to match subjects in experimental and comparison groups. The graphical visualization was used to validate the quality of the match before and after matching (see Figures 1–4). Figure 1 shows PSM estimates based on the common support area overlapped under the propensity scores between the experimental (blue bars) and comparison (red bars) groups, while green indicates off support. Figure 2 shows propensity scores on matched covariates, while Figures 3 and 4 show matched PS for experimental and treatment groups respectively. It was observed that most subjects in the experimental group had higher PS than those in the control group (Figures 3 and 4).

There was, however, evidence of a high level of common support. Of the 608 observations, only six did not align with the treatment category. Thus, they were dropped to create a balance between groups. The subsequent analyses were then based on 602 observations; 317 students were from the comparison group while 285 were from the experimental group, representing 52.66 percent and 47.34 percent respectively. By default, PSM analysis computes the average treatment effect on the treated (ATT) to compare the mean scores of students in the experimental and comparison groups. This estimate may not be accurate due to some teachers who might have defied (while others complied with) the request to implement the heuristic problem-solving approach after training (before intervention). Thus, using this estimate based on PSM analysis is not sufficient and could provide a wrong estimate attributed to the net effect due to the treatment administered during an intervention. Yet, it was premised that the estimated treatment effect would be unbiased and representative of the original sample. A more robust estimand called Difference in Difference (DID) was used to analyze the matched observations and hence estimate the treatment effect.

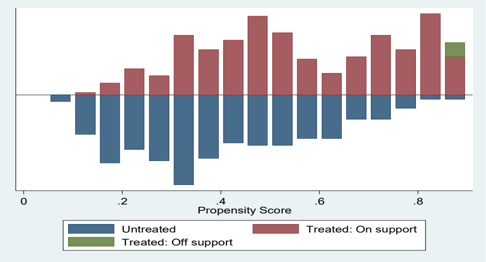

Figure 1: Showing Unmatched Propensity Scores
Source: Wakhata, Mutarutinya, and Balimuttajjo

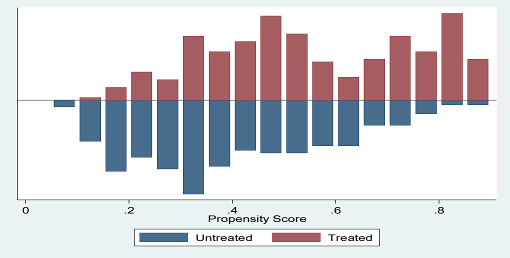

Figure 2: Showing Matched Propensity Scores
Source: Wakhata, Mutarutinya, and Balimuttajjo

Downloaded by Robert Wakhata on Thu Nov 24 2022 at 00:47:07 AM CST

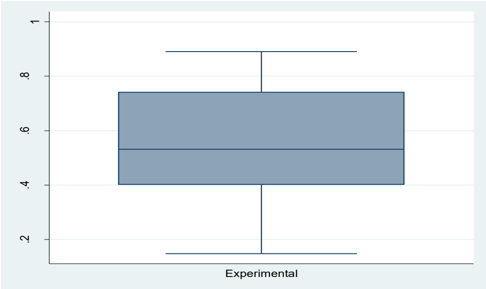

Figure 3: Matched Propensity Scores for Experimental Group
Source: Wakhata, Mutarutinya, and Balimuttajjo

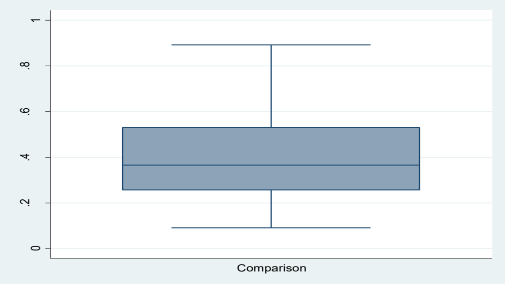

Figure 4: Matched Propensity Scores for Comparison Group
Source: Wakhata, Mutarutinya, and Balimuttajjo

Findings

Qualitative Findings through Classroom Observation

The findings point to the best learning practices concerning the effective application of the heuristic problem-solving approach. Although it was predicted that the learning in all the four experimental schools was uniform, different background characteristics influenced the learning environment. The heuristic problem-solving was adopted by all teachers from the experimental group although there were differences in its implementation. A uniform classroom observation scale was used to assess the learning across all the sampled schools. The research assistants conferenced with the specific mathematics teachers immediately after the lessons ended. This interaction was necessary for pointing out the strengths and weaknesses observed during the learning process, and together, the research assistants and the mathematics teachers suggested areas of lesson improvement. Specifically, comments were based on lesson preparation and organization, lesson presentation, the interaction between teachers and students, teachers' subject content knowledge and relevance, teachers' pedagogical knowledge, classroom management, and lesson assessment and evaluation.

Classroom observation and students' paper-and-pen responses provided opportunities for understanding patterns, their mathematical conjectures, and how they applied prior knowledge to find solutions to LP word problems. Students from the experimental group correctly applied and connected prior algebraic concepts of equations and inequalities in solving LP problems. The correct procedures were applied to find solutions to other general novel and non-routine LP problems. The HPSS fostered students' engagement, application of procedural knowledge, and conceptual knowledge during problem-solving and classroom instruction. The application of mathematics in students' daily lives was greatly emphasized to link the classroom application of LP to students' real-world scenarios. The varied solution sketches and alternative strategies provided clues for justifying, explaining, and supporting students' mathematical conceptual ideas and thinking. Consequently, students' responses were used as a springboard for making their conceptual generalizations, and for teachers' flexibility and variation of classroom instruction.

Generally, students from the treatment group exhibited active participation during problem-solving compared to their peers from the comparison group. It was also observed that students were free to engage in active discussions between teachers and amongst themselves (in small groups), and were able to apply the problem-solving strategies in solving related non-routine LP problems. This promoted collaborative learning for students' deeper and broader conceptual understanding. Individual students were also able to select, reflect, and justify solution strategies. Students' academic individual differences were effectively harmonized through group work. This consequently and further enhanced their academic and social relationships as they continued to solve LP and related mathematical challenging problems. Indeed, during an interaction with sampled

Downloaded by Robert Wakhata on Thu Nov 24 2022 at 00:47:07 AM CST

students to find out specific learning outcomes and the effect of the heuristic problem solving approach on students' achievement, one of the students noted that the heuristic problem-solving approach was more engaging. She echoed that sharing basic LP concepts amongst ourselves and later seeking clarifications from our mathematics teachers demystified the myth that LP word tasks are difficult to comprehend and pass. This particular student's posttest score had doubled (80%) relative to her pretest score (41%). This interview transcript supports quantitative findings.

Quantitative Findings through Propensity Score Matching and Difference in Difference

A Probit regression model was run (Table 5) to compute and compare the probability of each covariate for all subjects that received treatment to create a balance between treatment and comparison groups. The nearest neighborhood (NN) matching was used to create a balanced propensity score match between the two groups. Specifically, we used one-to-one matching. The numerical inspection of matched propensity scores was then used to compare the balance within covariates. Cohen's *d*, a robust numerical approach for estimating the effect size based on the mean comparison between the treated and the untreated subjects was used to compare standardized propensity score means ($d = 0.65$, CI [0.53, 0.76]). The effect size was found to be large enough to make substantial inferences based on recommendations from Cohen (1988) and Field (2009). The ratio of the variances between the treatment and the comparison groups on each covariate was also computed and was found to be approximately 1, which conforms to (Ho et al. 2007) recommendations. The visual inspection of the treated and comparison groups was done using quantile-quantile (QQ) plots and kernel density plots, which indicated a better balance and a satisfactory match between experimental and comparison groups. The graphical inspection further showed that the results were fairly normally distributed.

Table 1: The Mean Estimates of Pretest and Posttest Scores

	Mean	*Std. Err.*	*[95%_Conf]*	*[Interval]*
Pretest	44.21	0.76	42.73	45.70
Posttest	56.11	0.74	54.66	57.56

Source: Wakhata, Mutarutinya, and Balimuttajjo

Table 2: Student's Summary Statistics for Experimental and Comparison Groups

	Mean	*SD*
Comparison Group		
Pretest	42.79	18.59
Posttest	47.12	16.58
Experimental Group		
Pretest	45.76	18.61
Posttest	65.91	14.45

Source: Wakhata, Mutarutinya, and Balimuttajjo

Table 3: Showing Paired Sample t-Test

	Observations	*Mean 1*	*Mean 2*	*diff*	*Std. Err.*
Pretest—Posttest	291	45.763	65.91	-20.148	.895

Source: Wakhata, Mutarutinya, and Balimuttajjo

Evidence from Tables 1–3 show that students in the experimental group outperformed their peers in the comparison group before the matching. By gender, male students performed slightly better than their female counterparts (45.02, 58.13 vs 43.58, 54.54) in both pretest and posttest, respectively. Whereas there was a slight improvement in students' scores after an intervention, students studying from Central Uganda (44.89 vs 57.97) performed better than those studying in Eastern Uganda (43.48 vs 54.08) in pretest and posttest. On the other hand, students in boarding schools (47.54 vs 58.06) outperformed their peers in day schools (40.08 vs 53.69). By school ownership, students in private schools (46.18 vs 60.6) performed better than those in public schools (42.61 vs 52.45). Generally, there

Downloaded by Robert Wakhata on Thu Nov 24 2022 at 00:47:07 AM CST

was a slight improvement in the students' percentage scores in both tests by 4 percent and 20 percent for comparison and treatment groups respectively. It is, therefore, likely that the treatment administered had an impact on students' achievement in LP by 20 percent. A paired sample t-test shows that the results were statistically significant ($t(607) = 18.41$, $p = .000$), Moreover, the mean difference ($d = 0.65$) was large enough (Cohen 1988; Field 2009) to conclude that the heuristic problem-solving approach explained differences in students' achievement in LP.

No conclusion could be drawn based on the above analysis as multiple factors could have accounted for students' achievement in LP, and not necessarily the intervention that involved the application of the active learning through the heuristic problem-solving approach. By controlling for these confounding variables (gender, location of the school, school status, school ownership, and prior students' academic ability), the net effect and the magnitude of the teaching approach were further investigated. However, the slightly noticeable change in students' average scores from the experimental group probably suggested that the heuristic problem-solving approach could have influenced the students' achievement in LP. To confirm or reject the stated claim, the PSM analysis was used.

Table 4: Treatment-Effects Estimation

Posttest	*Coef.*	*Std. Err.*	*t-Value*	*p-Value*	*[95% Conf]*	*[Interval]*	*Sig.*
r1vs0.Group	12.546	1.095	11.46	0	10.4	14.692	***
Mean dependent var		56.112		SD dependent var		18.199	

Note: *** $p < 0.01$ ($r1$: Treatment group, $r0$: Comparison group)

Source: Wakhata, Mutarutinya, and Balimuttajjo

Table 5: Showing Probit Regression Predicting the Independent Variables

Group	*Coef.*	*Std. Err.*	*z*	*p > z*	*[95%Conf.]*	*[Interval]*
Pretest	−0.033	0.005	−6.080	0.000	−0.043	−0.022
Gender	0.111	0.110	1.010	0.313	−0.105	0.327
Age	0.018	0.057	0.320	0.747	−0.093	0.130
School location	0.205	0.109	1.880	0.061	−0.009	0.419
School status	0.392	0.112	3.500	0.000	0.172	0.611
School ownership	0.092	0.111	0.820	0.411	−0.127	0.310
Students' ability	1.460	0.166	8.810	0.000	1.135	1.784
_cons	−0.186	1.037	−0.180	0.858	−2.217	1.846

Variable Sample	*Treated*	*Controls*	*Difference*	*S.E.*	*T-stat*
Posttest unmatched	65.911	47.117	18.794	1.266	14.840
ATT	65.589	54.439	11.151	2.521	4.420

Assignment	*Off Support*		*On Support*		*Total*
Untreated	0		317	317	
Treated	6		285	291	
Total	6		602	608	

Source: Wakhata, Mutarutinya, and Balimuttajjo

Table 4 shows the estimated treatment effect of the heuristic problem-solving approach on the students' achievement in LP after adjusting for baseline characteristics. Before matching and using the pretest and posttest scores (Table 4), the average treatment effect on the treated (ATT) subjects was 12.55. However, it was still unclear whether or not the difference was due to an intervention. After running a Probit regression model, by default, the average treatment effect on the treated (ATET) was 11.15 (Table 5). It could not be confirmed at this point because PSM is used for matching similar covariates between the treatment and comparison groups. After matching, the potential outcome (PO) of the average scores for the treated and untreated groups were 63 and 50 percent respectively. Using the average treatment effect (ATE), the average scores for students in the treated group was 13 percent more than those in the untreated group. The average treatment effect on the treated (ATET) was 12.45 for the treatment group more than that of the comparison group. It was observed that the ATET after PSM is slightly higher than that before PSM (12.45 vs 11.15).

Downloaded by Robert Wakhata on Thu Nov 24 2022 at 00:47:07 AM CST

It is worth noting that a mere comparison of the pretest and posttest average differences for the treatment and comparison groups could not have controlled other background factors that trended between pretest and posttest. These factors could have accounted for changes in the students' achievement in LP and not necessarily due to the heuristic problem-solving approach. Notably, posttest neighborhoods for experimental and comparison groups may have had all kinds of differences as well and could not sufficiently explain whether or not the differences were due to the treatment. Although the values of ATT (12.55 and 11.15) were significant, the DID estimand (DID = 15.40, Table 6) supported qualitative results together with values of ATT to reject the null hypothesis (H0). In conclusion, there exists a substantial evidence that active learning through the heuristic problem-solving approach had a significant impact on the 11th grade students' learning outcomes of LP in the selected Ugandan secondary schools. Thus, the heuristic problem-solving approach increased the students' average scores by 15.40 percent (Table 6).

Table 6: The Average Difference in Difference (DID) Estimand

Group	*Posttest*	*Pretest*	*(Posttest) – (Pretest)*	*The Difference in Difference (DID)*
Comparison	47.12	42.79	4.32	15.40
Experimental	65.59	45.86	19.73	

Source: Wakhata, Mutarutinya, and Balimuttajjo

Discussion

This research examined the effect of active learning through the heuristic problem-solving approach on students' achievement in LP. A large body of research shows that inequalities and LP are intertwined in the creation of effective and efficient optimization strategies in Science, Technology, Engineering, and Mathematics (STEM) (Tsamir and Almog 2001). Therefore, appropriate learning approaches should be adopted/adapted to overcome students' challenges in algebra and LP in particular and consequently foster students' abilities. Specifically, equations and inequalities that are a basic requirement for students' understanding of LP should be adequately introduced and taught. If LP is not correctly introduced to secondary school students, the content and concepts may appear abstract with inherent difficulties. This may arise due to the incorrect application of basic algebraic concepts of equations and inequalities on the students' side and the instructional approaches applied by the teachers.

Based on the research findings, and after controlling for the background characteristics, the students from the treatment group performed significantly better than their peers from the comparison group by 15.40 percent (Table 6). Irrespective of the class size, this approach (if applied effectively) can be adopted in smaller cooperative learning groups to cater for students' academic differences and holistic learning (see Mukuka, Balimuttajjo, and Mutarutinya 2020). It was also observed that although there were no significant differences in students' average scores by gender, the majority of female students made several misconceptions and errors arising from inadequate knowledge of basic application of algebraic and symbolic representations. Students from boarding schools outperformed their peers from day schools. Moreover, it was observed that students from central Uganda (Mukono district) outperformed their colleagues from eastern Uganda (Mbale district). This trend had consistently been observed in the previous national examinations at UCE (UNEB 2016, 2018, 2019). Teachers from the experimental and comparison groups attributed this trend in performance to early syllabus coverage, motivation, and students' prior academic abilities at the time of admission to the eighth-grade (locally called senior one).

The findings in this study strongly suggest that the heuristic problem-solving approach had a significant effect on students' achievement in LP. The initial conventional approaches applied to the comparison group exhibited students' difficulties in comprehending specific mathematical theoretical principles as they learned. Some students who had developed a negative attitude toward LP rejuvenated and greatly improved their posttest scores when teachers from the experimental group adopted the heuristic problem-solving learning approach. Thus, the

Downloaded by Robert Wakhata on Thu Nov 24 2022 at 00:47:07 AM CST

application of the heuristic problem-solving approach enhanced students' cognitive and affective domains in basic algebraic content that later facilitated and enhanced their mathematical conceptual understanding, procedural fluency, and competency in mathematics and related LP tasks. According to Julius, Abdullah, and Suhairom (2018), students' success in mathematics word problems is hampered by their lack of prior conceptual understanding combined with a negative attitude toward solving basic algebraic questions.

Kenney et al. (2020) and Khoshaim (2020) noted that mathematics word problems are challenging for most students to comprehend. The challenges faced by students in LP, "the cousin" of mathematics word problems are, therefore, not by chance. Based on the above studies and other empirical findings, this study highly recommends teachers to adopt the heuristic problem-solving approach to enhance students' critical thinking skills, conceptual understanding, procedural fluency, strategic competence, adaptive reasoning, and productive disposition when solving mathematics tasks and LP in particular.

Previous research has shown that LP word problems and/or related topics are not only difficult for students to understand, but also difficult to teach (Awofala 2014; Kenney et al. 2020; Molina et al. 2017; Verikios and Farmaki 2010; Goulet-Lyle, Voyer, and Verschaffel 2020; Verschaffel et al. 2020; Pongsakdi et al. 2019, 2020). Different variables account for students' challenges in learning mathematics word problems. The interviews conducted with some students from the comparison group revealed students' inability to effectively comprehend mathematics word problems which leads to alterations in procedural comprehension and competency. It was observed that part of the learning challenges stem from students' academic background, prior conceptual understanding, and students' attitudes toward mathematics. This study's findings are in agreement with those of Ahmad, Tarmizi, and Nawawi (2010), and Kenney et al. (2020). Thus, teachers should use instructional approaches that integrate students' learning difficulties to overcome the aforementioned challenges. The qualitative interview results with some students and teachers from the experimental group also revealed that the heuristic problem-solving approach positively impacted students' achievement in LP.

To overcome learning challenges, teachers can effectively teach, guide, and counsel students to build a positive attitude toward mathematics. Expert teachers may be allowed to share their insights about the best strategies and approaches for increasing and fostering students' mathematical abilities through seminars and workshops. Regardless of gender disparities, school location, school status, or other background characteristics, teachers should collectively guide students to set and realize their goals in mathematical problem-solving beyond classroom activities. How and when functions, equations, and inequalities should be presented is a major concern during classroom instruction. Which learning approach should be used first, and how should concepts be presented? The graphical method of solving LP problems was mainly stressed in this study. However, if the essential concepts are misunderstood, students may be unable to effectively answer a particular LP problem. Teachers should, therefore, review and cover the basic algebraic concepts that may aid or hinder students' grasp and subsequent learning of LP.

Finally, because the goal of this study was to test the research hypothesis of the effect of the heuristic problem-solving approach on students' achievement in LP, it was more quantitative with some supportive qualitative findings. The interview transcripts were not thoroughly scrutinized to elicit students' difficulties in learning LP. To make inferences, we mostly relied on the students' pretest and posttest scores. However, we may have presented and analyzed students' vignettes with evidence of students' specific preconceptions, misconceptions, and errors made to gain a deeper and broader grasp and insight of their LP abilities. As a result, the qualitative findings could be interpreted in a variety of ways. This is something we fully acknowledge, and we look forward to hearing from future researchers.

Downloaded by Robert Wakhata on Thu Nov 24 2022 at 00:47:07 AM CST

Conclusion

This study sought to examine whether or not there was a statistically significant change in students' average scores in LP problems after being exposed to and taught using the heuristic problem-solving instructional approach. PSM method was used to account for selection bias after matching experimental to the comparison group. The results of this study showed that the null hypothesis was rejected in favor of the alternative hypothesis. The posttest average scores of students from the experimental group were significantly higher than those in the comparison group. We can deduce that the heuristic problem-solving approach received by students in the experimental group made a significant change in students' average marks. This, therefore, revealed the effectiveness of the intervention approach in terms of students' conceptual understanding, procedural fluency, strategic competence, adaptive reasoning, and productive disposition. It was further observed that most students from the experimental group demonstrated proficiency in problem-solving in terms of their ability in solving LP tasks. The heuristic problem-solving learning approach can, therefore, be adopted during the learning of mathematics in secondary schools and beyond to help students enhance their conceptual proficiency.

While this study demonstrates the effect of heuristic problem-solving and complements previous empirical findings, there are some limitations. First, the study relied on observational data meaning that the unobserved covariates (e.g., students' motivation, teachers' motivation, teachers' experience, teachers' pedagogical content knowledge, learning materials, students' cognitive levels, etc.,) that may have influenced students' achievement in LP were not considered. Second, the schools were purposively sampled and were not a random sample of secondary schools in Uganda. The findings may not explicitly explain a representative students' population in Uganda. Therefore, caution must be exercised when generalizing our findings. We recommend further studies in analyzing students' national examination answer scripts on LP problems, and in different contexts and settings, based on the schools' baseline characteristics. To do this, there is a need to train all teachers through continuous professional development courses to collectively and effectively implement the heuristic problem-solving approach and consequently evaluate its effect on students' mathematical proficiency.

Acknowledgement

This research is part of the PhD thesis financially supported by the African Centre of Excellence for Innovative Teaching and Learning Mathematics and Science (ACEITLMS), University of Rwanda, College of Education. We are grateful for the useful information provided by the teachers and students from the study sample. While we acknowledge financial support from ACEITLMS, the Centre was not involved in identifying suitable study approach, design, methods of data collection and analysis, findings, conclusions, and recommendations.

REFERENCES

Abdelsamad, Refat, and Abouelgheat Kandeel. 2021. "Learners' Mathematics Proficiency Levels on PISA 2018: A Comparative Study." *International Journal of Instruction* 14 (3): 393–416. https://doi.org/10.29333/iji.2021.14323a.

Abu Bakar, Mohamad Ariffin Abu, and Norulhuda Ismail. 2020. "Mathematical Instructional: A Conceptual of Redesign of Active Learning with Metacognitive Regulation Strategy." *International Journal of Instruction* 13 (3): 633–648. https://doi.org/10.29333/iji.2020.13343a.

Ahmad, Azizah, Rohani Ahmad Tarmizi, and Mokhtar Nawawi. 2010. "Visual Representations in Mathematical Word Problem Solving among Form Four Students in Malacca." *Procedia—Social and Behavioral Sciences* 8 (2010): 356–361. https://doi.org/10.1016/j.sbspro.2010.12.050.

Downloaded by Robert Wakhata on Thu Nov 24 2022 at 00:47:07 AM CST

Alex, Jogymo, and Kuttikkattu J. Mammen. 2018. "Students' Understanding of Geometry Terminology through the Lens of Van Hiele Theory." *Pythagoras* 39 (1): 1–8. https://doi.org/10.4102/pythagoras.v39i1.376.

Allsopp, David H., Maggie M. Kyger, and Lou Ann H. Lovin. 2007. *Teaching Mathematics Meaningfully: Solutions for Reaching Struggling Learners.* 2nd ed. Baltimore: Brookes Publishing.

Almog, Nava, and Bat-Sheva Ilany. 2012. "Absolute Value Inequalities: High School Students' Solutions and Misconceptions." *Educational Studies in Mathematics* 81 (3): 347–364. https://doi.org/10.1007/s10649-012-9404-z.

Angraini, Lilis Marina, and Astri Wahyuni. 2020. "The Effect of Concept Attainment Model on Mathematical Critical Thinking Ability." *International Journal of Instruction* 14 (1): 727–742. https://doi.org/10.29333/IJI.2021.14144A.

Apkarian, Naneh, Charles Henderson, Marilyne Stains, Jeffrey Raker, Estrella Johnson, and Melissa Dancy. 2021. "What Really Impacts the Use of Active Learning in Undergraduate STEM Education? Results from a National Survey of Chemistry, Mathematics, and Physics Instructors." *PLoS ONE* 16 (2): 1–15. https://doi.org/10.1371/journal.pone.0247544.

Awofala, Adeneye O. A. 2014. "Examining Personalisation of Instruction, Attitudes toward and Achievement in Mathematics Word Problems among Nigerian Senior Secondary School Students." *International Journal of Education in Mathematics, Science and Technology* 2 (4): 273–288.

Bazzini, Luciana, and Pessia Tsamir. 2004. "Algebraic Equations and Inequalities: Issues for Research and Teaching." Paper presented at the *Proceedings of the 28th Conference of the International Group for the Psychology of Mathematics Education (PME)*, Bergen, NO, July 2004.

Budak, Ayfer. 2015. "The Impact of a Standards-Based Mathematics Curriculum on Students' Mathematics Achievement: The Case of Investigations in Number, Data, and Space." *Eurasia Journal of Mathematics, Science and Technology Education* 11 (6): 1249–1264. https://doi.org/10.12973/eurasia.2015.1377a.

Cohen, Jacob. 1988. *Statistical Power Analysis for the Behavioral Sciences*. 2nd ed. New York: Academic Press.

Creswell, John W. 2014. *Research Design: Qualitative, Quantitative, and Mixed Methods Approaches.* 4th ed. Thousand Oaks, CA: SAGE Publications.

Creswell, John W., and Clark Vicki L. Plano. 2018. *Designing and Conducting Mixed Methods Research.* 3rd ed. Los Angeles: SAGE.

De Jesus, Cristina Cirino, Cristina Márcia de C. T. Cyrino, and Hélia Oliveira. 2015. "Tasks Analysis as a Means to Reflect and (Re)think the Pedagogical Practice of Teachers Who Teach Mathematics." Paper presented at the CERME 9-Ninth Congress of the European Society for Research in Mathematics Education, Charles University, Faculty of Education; ERME, Prague, Czech Republic, February 2015: 2818–2824.

Fan, Xitao, and Dana L. Nowell. 2015. "Using Propensity Score Matching in Educational Research." *Gifted Child Quarterly* 55 (1): 74–79. https://doi.org/10.1177/0016986210390635.

Feden, Preston D., and Robert Mark Vogel. 2003. "Methods of Teaching: Applying Cognitive Science to Promote Student Learning." *International Journal of Science and Technology Education Research* 11 (1): 1–13.

Field, Andy. 2009. *Discovering Statistics Using SPSS*. 3rd ed. London: SAGE Publications.

Fraenkel, Jack R., Norman E. Wallen, and Helen H. Hyun. 2011. *How to Design and Evaluate Research in Education.* 8th ed. New York: McGraw-Hill.

Downloaded by Robert Wakhata on Thu Nov 24 2022 at 00:47:07 AM CST

Goulet-Lyle, Marie-Pier, Dominic Voyer, and Lieven Verschaffel. 2020. "How Does Imposing a Step-by-Step Solution Method Impact Students' Approach to Mathematical Word Problem Solving?" *ZDM—Mathematics Education* 52 (1): 139–149. https://doi.org/10.1007/s11858-019-01098-w.

Ho, Daniel E., Kosuke Imai, Gary King, and Elizabeth A. Stuart. 2007. "Matching as Nonparametric Preprocessing for Reducing Model Dependence in Parametric Causal Inference." *Political Analysis* 15 (3): 199–236. https://doi.org/10.1093/pan/mpl013.

Jonassen, David H. 2011. *Learning to Solve Problems. A Handbook for Designing Problem-Solving Learning Environments.* NewYork: Routledge.

Julius, Elizabeth, Abdul Halim Abdullah, and Nornazira Suhairom. 2018. "Attitude of Students towards Solving Problems in Algebra: A Review of Nigeria Secondary Schools." *Journal of Research & Method in Education* 8 (1): 26–31.

Jupri, Al, and Paul Drijvers. 2016. "Student Difficulties in Mathematizing Word Problems in Algebra." *Eurasia Journal of Mathematics, Science and Technology Education* 12 (9): 2481–2502. https://doi.org/10.12973/eurasia.2016.1299a.

Juras, Randall. 2016. "Estimates of Intraclass Correlation Coefficients and Other Design Parameters for Studies of School-Based Nutritional Interventions." *Evaluation Review* 40 (4): 314–333. https://doi.org/10.1177/0193841X16675223.

Kenney, Rachael, Tuyin An, Sung-Hee Kim, Nelson A. Uhan, Ji Soo Yi, and Aiman Shamsul. 2020. "Linear Programming Models: Identifying Common Errors in Engineering Students' Work with Complex Word Problems." *International Journal of Science and Mathematics Education* 18 (4): 635–655. https://doi.org/10.1007/s10763-019-09980-5.

Khoshaim, Heba Bakr. 2020. "Mathematics Teaching Using Word-Problems: Is It a Phobia!?" *International Journal of Instruction* 13 (1): 855–868. https://doi.org/10.29333/iji.2020.13155a.

Kilpatrick, Jeremy, Jane Swafford, and Bradford Findell. 2001. *Adding It Up*: *Helping Children Learn Mathematics.* Washington, DC: National Academy Press.

Koo, Terry K., and Mae Y. Li. 2016. "A Guideline of Selecting and Reporting Intraclass Correlation Coefficients for Reliability Research." *Journal of Chiropractic Medicine* 15 (2): 155–163. https://doi.org/10.1016/j.jcm.2016.02.012.

Lane, Forrest C., and Gibbs SherRhonda. 2015. "Propensity Score Analysis: A Secondary Data Analysis of Work-Life Policy and Performance Outcomes." *Advances in Developing Human Resources* 17 (1): 102–116. https://doi.org/10.1177/1523422314559809.

Lesh, Richard, Mark Hoover, Bonnie Hole, Anthony Kelly, and Thomas Post. 2000. "Principles for Developing Thought-Revealing Activities for Students and Teachers." In *Research Design in Mathematics and Science Education*, 591–646. Mahwah, NJ: Lawrence Erlbaum.

Makonye, Judah, and Mhonda Shingirayi. 2014. "The Obstacles Faced by the Learners in the Learning of Quadratic Inequalities." *Mediterranean Journal of Social Sciences* 5 (27): 716–725. https://doi.org/10.5901/mjss.2014.v5n27p716.

Molina, Marta, Susana Rodríguez-Domingo, María Consuelo Cañadas, and Encarnación Castro. 2017. "Secondary School Students' Errors in the Translation of Algebraic Statements." *International Journal of Science and Mathematics Education* 15 (6): 1137–1156. https://doi.org/10.1007/s10763-016-9739-5.

Mukuka, Angel, Sudi Balimuttajjo, and Védaste Mutarutinya. 2020. "Applying the Solo Taxonomy in Assessing and Fostering Students' Mathematical Problem-Solving Abilities." In *Proceedings of the 28th Annual Conference of the Southern African Association for Research in Mathematics, Science and Technology Education*, January 2020, edited by P. Vale, L. Westaway, Z. Nhase, and I. Schudel, 104–112. Eastern Cape, South Africa: SAARMSTE.

Downloaded by Robert Wakhata on Thu Nov 24 2022 at 00:47:07 AM CST

Mushlihah, Rohmah, and Sutiarso Sugeng. 2018. "Analysis Problem Solving in Mathematical Using Theory Newman." *Eurasia Journal of Mathematics, Science and Technology Education* 14 (2): 671–681. https://doi.org/10.12973/ejmste/80630.

NCDC (National Curriculum Development Centre). 2008. *The Ministry of Education and Sports. Mathematics Teaching Syllabus for Uganda Certificate of Education*. Kampala, UG: NCDC.

NCDC (National Curriculum Development Centre). 2018. *The Ugandan Lower Secondary School Curriculum: Mathematics Syllabus*. Kampala, UG: NCDC.

NCTM (National Council of Teachers of Mathematics). 2014. *Principles to Actions: Ensuring Mathematical Success for All.* Reston, VA: NCTM.

Ofori-kusi, Daniel, and David Mogari. 2017. "Effects of a Problem-Solving Heuristic Instructional Approach in the Learning of Algebra in Grade 6." Paper presented at the Proceedings of the 25th Annual Conference of the Southern African Association for Research in Mathematics, Science and Technology Education, Central University of Technology, SAARMSTE, Bloemfontein, SA, November 2017: 26–35.

Oosterwijk, Pieter R., L. Andries van der Ark, and Klaas Sijtsma. 2019. "Using Confidence Intervals for Assessing Reliability of Real Tests." *Assessment* 26 (7): 1207–1216. https://doi.org/10.1177/1073191117737375.

Polya, G. 2004. *How to Solve It: A New Aspect of Mathematical Method.* 2th ed. New York: Princeton University Press. https://doi.org/10.2307/j.ctvc773pk.6.

Pongsakdi, Nonmanut, Anu Kajamies, Koen Veermans, Kalle Lertola, Marja Vauras, and Erno Lehtinen. 2020. "What Makes Mathematical Word Problem Solving Challenging? Exploring the Roles of Word Problem Characteristics, Text Comprehension, and Arithmetic Skills." *ZDM—Mathematics Education* 52 (1): 33–44. https://doi.org/10.1007/s11858-019-01118-9.

Pongsakdi, Nonmanut, Eero Laakkonen, Teija Laine, Koen Veermans, M. Hannula-Sormunen, and Erno Lehtinen. 2019. "The Role of Beliefs and Motivational Variables in Enhancing Word Problem Solving." *Scandinavian Journal of Educational Research* 63 (2): 179–197. https://doi.org/10.1080/00313831.2017.1336475.

Quintero, Ana Helvia, and Héctor Rosario. 2016. *Math Makes Sense! A Constructivist Approach to the Teaching and Learning of Mathematics*. London: Imperial College Press.

Rosenbaum, Paul R., and Donald B. Rubin. 2007. "Reducing Bias in Observational Studies Using Subclassification on the Propensity Score." *Journal of the American Statistical Association* 79 (387): 516–524. https://doi.org/10.2307/2288398.

Samuelsson, Joakim. 2010. "The Impact of Teaching Approaches on Students' Mathematical Proficiency in Sweden." *International Electronic Journal of Mathematics Education* 5 (2): 61–78.

Shafer, Mary C., Lesley Wagner, and Jon Davis. 1997. *Classroom Observation Scale: (Mathematics in Context Longitudinal/Cross-Sectional Study)*. Working Paper No. 6). Madison, WI: University of Wisconsin.

Shikuku, Beatrice Nakhanu, and Maurice Musasia Amadalo. 2015. "Problem-Based Learning Technique and Its Effect on Acquisition of Linear Programming Skills by Secondary School Students in Kenya." *Journal of Education and Practice* 6 (20): 68–75.

Smith, Margaret S., and Mary Kay Stein. 1998. "Selecting and Creating Mathematical Tasks: From Research to Practice." *Mathematics Teaching in the Middle School* 3 (5): 344–350.

Stein, Mary Kay, Barbara W. Grover, and Marjorie Henningsen. 1996. "Building Student Capacity for Mathematical Thinking and Reasoning: An Analysis of Mathematical Tasks Used in Reform Classrooms." *American Educational Research Journal* 33 (2): 455–488. https://doi.org/10.3102/00028312033002455.

Stein, Mary Kay, Margaret Schwan Smith, Majorie A. Henningsen, and Edward A. Silver. 2000. *Implementing Standards-Based Math Instruction: A Casebook for Professional Development*. New York: Teachers College Press.

Downloaded by Robert Wakhata on Thu Nov 24 2022 at 00:47:07 AM CST

Suh, Jennifer M. 2007. “Tying It All together. Classroom Practices That Promote Mathematical Proficiency for All Students.” *Teaching Children Mathematics* 14 (3): 163–169. https://doi.org/10.5951/TCM.14.3.0163.

Ting, Fridolin Sze Thou, Wai Hung Lam, and Ronnie Homi Shroff. 2019. “Active Learning via Problem-Based Collaborative Games in a Large Mathematics University Course in Hong Kong.” *Education Sciences* 9 (3): 172. https://doi.org/10.3390/educsci9030172.

Tsamir, Pessia, and Luciana Bazzini. 2004. “Consistencies and Inconsistencies in Students’ Solutions to Algebraic ‘Single-Value’ Inequalities.” *International Journal of Mathematical Education in Science and Technology* 35 (6): 793–812. https://doi.org/10.1080/00207390412331271357.

Tsamir, Pessia, and Nava Almog. 2001. “Students’ Strategies and Difficulties: The Case of Algebraic Inequalities.” *International Journal of Mathematical Education in Science and Technology* 32 (4): 513–524. https://doi.org/10.1080/00207390110038277.

UNEB (Uganda National Examinations Board). 2016. *Report on Work of Uganda Certificate of Examinations Candidates.* Kampala, UG: UNEB.

UNEB (Uganda National Examinations Board). 2018. *Report on Work of Uganda Certificate of Examinations Candidates.* Kampala, UG: UNEB.

UNEB (Uganda National Examinations Board). 2019. *Report on Work of Uganda Certificate of Examinations Candidates.* Kampala, UG: UNEB.

UNEB (Uganda National Examinations Board). 2020. *Report on Work of Uganda Certificate of Examinations Candidates.* Kampala, UG: UNEB.

Verikios, Petros, and Vassiliki Farmaki. 2010. “From Equation to Inequality Using a Function-Based Approach.” *International Journal of Mathematical Education in Science and Technology* 41 (4): 515–530. https://doi.org/10.1080/00207390903564611.

Verschaffel, Lieven, Stanislaw Schukajlow, Jon Star, and Dooren Wim Van. 2020. “Word Problems in Mathematics Education: A Survey.” *ZDM—Mathematics Education* 52 (1): 1–16. https://doi.org/10.1007/s11858-020-01130-4.

ABOUT THE AUTHORS

Robert Wakhata: PhD Student in Mathematics Education, African Centre of Excellence for Innovative Teaching and Learning Mathematics and Science (ACEITLMS), University of Rwanda, College of Education (UR-CE), Rwamagana, Rwanda

Dr. Védaste Mutarutinya: Senior Lecturer of Mathematics, African Centre of Excellence for Innovative Teaching and Learning Mathematics and Science (ACEITLMS), University of Rwanda, College of Education (UR-CE), HEC Board Member–Rwanda, Rwamagana, Rwanda

Dr. Sudi Balimuttajjo: Senior Lecturer of Mathematics Education, Department of Educational Foundations and Psychology, Mbarara University of Science and Technology, Mbarara City, Uganda

Downloaded by Robert Wakhata on Thu Nov 24 2022 at 00:47:07 AM CST

The International Journal of Science, Mathematics and Technology Learning is one of ten thematically focused journals in the collection of journals that support The Learner Research Network—its journals, book series, conference, and online community.

The journal offers studies of best practices in teaching and learning science, mathematics, and technology.

As well as articles of a traditional scholarly type, this journal invites presentations of practice—including documentation of curricular practices and exegeses of the effects of those practices.

The International Journal of Science, Mathematics and Technology Learning is a peer-reviewed, scholarly journal.

ISSN 2327-7971

Downloaded by Robert Wakhata on Thu Nov 24 2022 at 00:47:07 AM CST
