# Supplementary material for: Dataset on the relationship between students’ attitude towards, and performance in mathematics word problems, mediated by active learning heuristic problem-solving approach
Source: Data Brief. 2023 Mar 14;48:109055. doi: 10.1016/j.dib.2023.109055 (PMC10051018; doi:10.1016/j.dib.2023.109055)
Supplement: Supplementary file 1 [file mmc1.zip › Supplementary material for DIB/Focus Group Prompts.pdf]

FOCUS GROUP PROMPTS

1. What teaching techniques helped to improve your performance and attitude towards learning linear programming? this might be thought of in terms of:
   - Enjoyment
   - Anxiety
   - Confidence
   - Motivation to pursue further courses in mathematics
   - Value of linear programming for your personal or professional development

2. What teaching techniques hindered your performance and attitude towards learning linear programming? this might be thought of in terms of:

- Enjoyment
- Anxiety
- Confidence
- Motivation to pursue further courses in mathematics
- Value of linear programming for your personal or professional development

3. In your opinion, what do you think can be done to improve students' performance and attitude towards learning linear programming in secondary schools in Uganda? this might be thought of in terms of improving students':
   - Enjoyment
   - Anxiety
   - Confidence
   - Motivation to pursue further courses in mathematics
   - Value of linear programming for your personal or professional development

Thank you for your time, patience and voluntary participation in this study!
