# Supplementary material for: Dataset on the relationship between students’ attitude towards, and performance in mathematics word problems, mediated by active learning heuristic problem-solving approach
Source: Data Brief. 2023 Mar 14;48:109055. doi: 10.1016/j.dib.2023.109055 (PMC10051018; doi:10.1016/j.dib.2023.109055)
Supplement: Supplementary file 1 [file mmc1.zip › Supplementary material for DIB/Interview Guide.pdf]

INTERVIEW GUIDE

1. What challenges do you face during the learning of inequalities and linear programming?

2. Which techniques do teachers use when teaching inequalities and linear programming?

3. Which aspects (concepts) of inequalities and linear programming were most problematic?

4. How do teachers help you to overcome challenges in inequalities and linear programming?

5. How do teachers identify learners with challenges in inequalities and linear programming?

6. What else do they do to help learners understand inequalities and linear programming?

7. In your own opinion, what should be done to improve the learning of inequalities and linear programming in your school?

Thank you for your patience, contributions and voluntary participation in this study!.
