# Supplementary material for: Dataset on the relationship between students’ attitude towards, and performance in mathematics word problems, mediated by active learning heuristic problem-solving approach
Source: Data Brief. 2023 Mar 14;48:109055. doi: 10.1016/j.dib.2023.109055 (PMC10051018; doi:10.1016/j.dib.2023.109055)
Supplement: Supplementary file 1 [file mmc1.zip › Supplementary material for DIB/Introduction and Informed Consent.pdf]

Letter of Introduction to Participants and Informed Consent Form

Dear Participant,
I am a Ph.D. Student at the College of Education, University of Rwanda, Rwanda. Currently, I am conducting research in mathematics on the topic: "Effect of Active Learning through the Heuristic Method on Students' Achievement and Attitude towards Linear Programming in Ugandan Secondary Schools". Linear programming (LP) is one of the topics within the Ugandan lower secondary school curriculum where students experience learning obstacles, and there is little or no research that has examined the effect of different heuristics on students' achievement and attitude towards this topic. Therefore, the findings arising from this study will enhance the learning of LP and mathematics in general.

The following statement describes the purpose, procedures, benefits, risks, and discomforts of this study. Please, I strongly encourage you to read it carefully before accepting or declining to participate in this study. Note that it is your right to participate, refuse or withdraw from this research at any time if deemed necessary. This research passed through an internal review collegial process and was approved by the Directorate of Research and Innovation of the University of Rwanda, College of Education.

Explanation of Procedures: This study examines the effect of active learning through the heuristic method on students' achievement and attitude towards linear programming in Ugandan secondary schools. I am conducting this study to learn more about the teaching and learning of LP in Ugandan secondary schools. This research will target the 11th grade (locally called senior four) students and teachers of mathematics from sampled secondary schools in Uganda. Participation in this study shall involve the completion of tests, questionnaire items, face-to-face interviews, focus group discussions and being observed. This may last for approximately 30 minutes-1hour. Interviews about the learning of LP will be: (1) conducted at your school (2) film/photographed (3) audio or videotaped, and later will be transcribed for the main purpose of data analysis, interpretation, and writing of a Ph.D. Thesis and/or journal articles.

Risks and Discomforts: There are no foreseeable/potential physical, social or economic risks or discomforts that are anticipated to occur/arise from your participation in this research. However, in case any participant experiences adverse effects arising from this study, the principal researcher or his research team must be contacted immediately.

Benefits: The anticipated benefit(s) of your participation in this study is the opportunity to discuss the best strategies to enhance the learning of LP in Ugandan secondary schools. Participation in this study might not directly benefit you. However, the information that you may provide will be useful to secondary school teachers and students in improving classroom practices.

Confidentiality: All information obtained from this study shall be stored in secure premises (locked, secure, filing cabinet) and guarded with utmost confidentiality, and will be used for a Ph.D. Thesis write-up and journal publications only. Therefore, your identity or that of all individuals in the focus group will remain anonymous, and your name (and any other identifying details) or that of your institution will not be disclosed on the surveys or interview transcripts during dissemination of research findings. Instead, participants' identity will be translated into ID

codes. Only the principal researcher or his team will have access to the study data premises and hear/view the recorded information. The recording gadgets or stored information for instance on tapes will be destroyed at the completion of this study.

Withdrawal without Prejudice: Participation in this study is strictly voluntary. In case you choose not to participate, you will not be penalized. You may refuse to answer any (some) question(s) asked, participate in any activity, discontinue participation or withdraw consent at any time by notifying me or my research team. Upon your request to withdraw, all information collected by then will be erased or destroyed.

Further Questions and Follow-up: For additional information or any inquiries concerning this study before, during, or after data collection, do not hesitate to ask or contact me directly, by phone or email. For more details, you may also contact the head of Research African Centre of Excellence for Innovative Teaching and Learning Mathematics and Science (ACEITLMS) based at University of Rwanda, College of Education (UR-CE) via www.ur.ac.rw.
Thank you for your cooperation.
Yours sincerely,

………………….

Robert Wakhata
Ph.D. Student (Mathematics Education).
University of Rwanda, College of Education, Rwanda. Telephone: +250785333300 / +256 779 038 552 / +256 701 692 986 Email: rwakhata@gmail.com

Consent to Participate in this Research
I have read, and understood the purpose of this study. I am satisfied with all the information provided on this form, and I willingly consent to volunteer to be part of this study. I understand that my responses will be: filmed, recorded on audio/video tape, completely confidential, anonymous, and treated with academic integrity. I also have the right to withdraw from this study at any time without prejudice if deemed necessary. I have received and completed a copy of the informed consent form to provide feedback and additional information to this study.

Participants aged 18 and parents (legal guardians) of participants below 18 years
By signing below, I declare that I understand the researcher's explanation of the purpose, scope, objectives, methods of data collection, and benefits of this research including the integrity of data to be collected by the principal researcher and/or his research team.

Consent of participants aged 18 and above
Respondents' name (optional): ……………………………………Sign…............Date................
Contact address: ……………………………………………………………………………………
E-mail address: …………………………………………Telephone number(s): ………………….

Consent of Parents or legal guardians
Parents' or legal guardians' name (optional): ……………………...........Sign….........Date.........
Contact address: ……………………………………………………………………………………
E-mail address: ……………………………………………Telephone number(s): ……………….
