# Supplementary material for: Dataset on the relationship between students’ attitude towards, and performance in mathematics word problems, mediated by active learning heuristic problem-solving approach
Source: Data Brief. 2023 Mar 14;48:109055. doi: 10.1016/j.dib.2023.109055 (PMC10051018; doi:10.1016/j.dib.2023.109055)
Supplement: Supplementary file 1 [file mmc1.zip › Supplementary material for DIB/LIPAT Pre.pdf]

**LINEAR PROGRAMMING ACHIEVEMENT TEST (PRE-TEST)**

**Instructions:**
**Answer ALL the questions**
**Time allowed: 1 Hour, 20 minutes**

1. (a) Solve the inequality $6 \geq 7 - x \geq 2$ and state all the integral values of **x**:

   (b) By writing the equation $3y - 2x - 6 = 0$ in the form $y = mx + c$, find the gradient.

   (c) If the line $3y - 2x - 6 = 0$ crosses the $x - axis\ at\ \boldsymbol{A}\ and\ the\ y - axis\ at\ \boldsymbol{B},$ write down the coordinates of $\boldsymbol{A}$ and $\boldsymbol{B}$.

   (d) Peter, a trader has Shs.250,000. He buys boxes of books at Shs.25,000 each and boxes of candles at Shs.10.000 each. The money spent on books is at least 50,000 more than that spent on candles. He buys at least 5 boxes of books and at least 7 boxes of candles. Write down four inequalities to represent this information.

2. On the same axes, and using the scale of **1**cm:**2**units on ***x***-axis and **1**cm:**1**unit on ***y***-axis;

   (a) Draw and label the graphs of the following lines:
   (i) $x + y = 4$ (ii) $x - y = 4$ (iii) $y = -3x$

   (b) By shading the unwanted regions, show clearly the feasible region $\boldsymbol{R}$ which satisfies the following inequalities: (i) $x + y \leq 4$ (ii) $y \geq x - 4$ (iii) $y > -3x$

   (c) Write down the values of ***x*** and ***y*** which gives the maximum values of $x - 3y$

3. By shading the unwanted regions, show the feasible region **R** satisfying the following inequalities: (a) $y < x + 1$ (b) $x + y > 5$ (c) x ≤ 4 hence, minimize $(x - 4y)$.

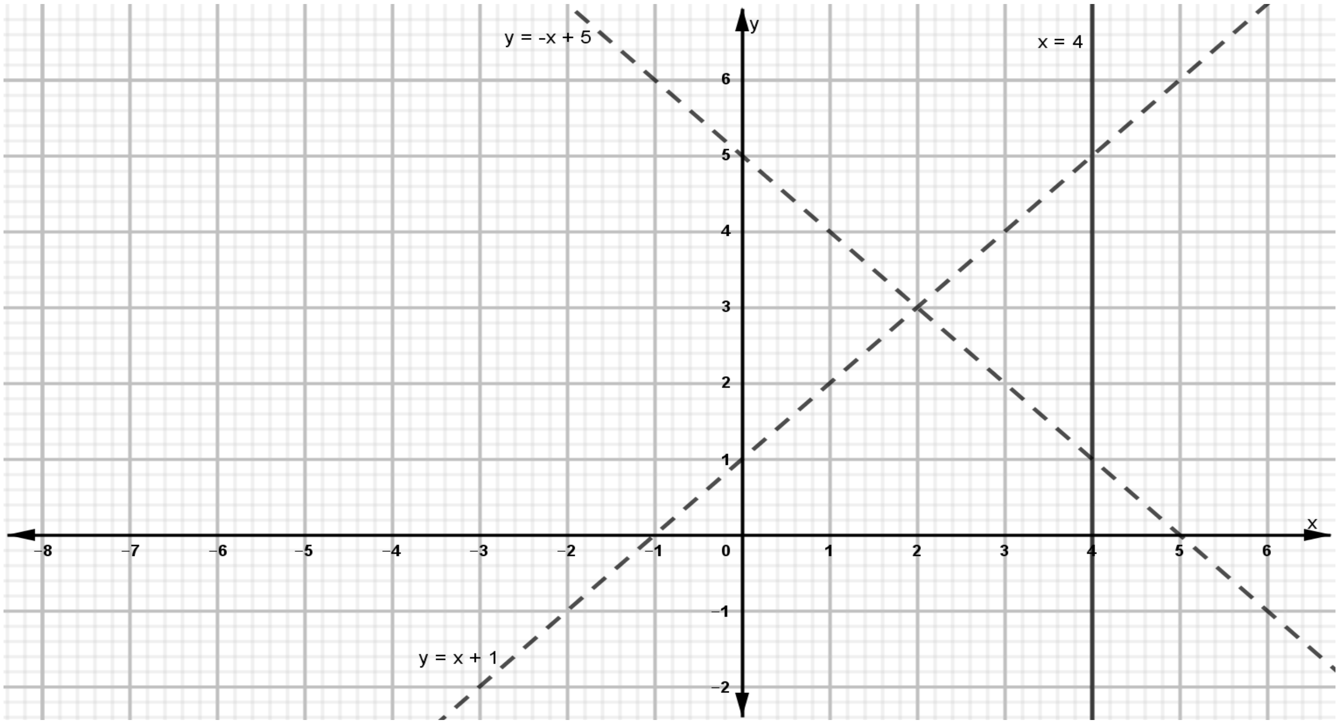


**The end**
