# Supplementary material for: Dataset on the relationship between students’ attitude towards, and performance in mathematics word problems, mediated by active learning heuristic problem-solving approach
Source: Data Brief. 2023 Mar 14;48:109055. doi: 10.1016/j.dib.2023.109055 (PMC10051018; doi:10.1016/j.dib.2023.109055)
Supplement: Supplementary file 1 [file mmc1.zip › Supplementary material for DIB/Research recommendation.pdf]

UNIVERSITY of RWANDA

COLLEGE OF EDUCATION

RESEARCH AND INNOVATION OFFICE

Rukara, 26th October, 2020
Ref: 03/DRI-CE/061(a)/EN/gi/2019

The Permanent Secretary
Ministry of Education and Sports, Uganda
P.o.Box 7063
Kampala-Uganda

Dear Permanent Secretary,

Re: Recommendation for Mr. Robert Wakhata to conduct a research in Uganda

I am pleased to confirm that Mr. Robert Wakhata is currently a doctoral student at the African Centre of Excellence for Innovative Teaching and Learning Mathematics and Sciences (ACEITLMS) hosted by the University of Rwanda-College of Education (UR-CE). He is conducting research entitled "**Effect of Active Learning through the Heuristic Method on students' Achievement and Attitude towards Linear Programming in Ugandan Secondary Schools.**" This research will involve mathematics teachers and, senior four (S4) registered students from secondary schools (both government and Private) for the 2020 school year. His fieldwork time frame is between October 2020 and February 2021.

Mr Wakhata's research project passed through an internal collegial ethical process. Thus, the University of Rwanda-College of Education: Directorate of Research and Innovation confirms that his research adheres to ethical standards and principles. Considering the contribution of this study in the promotion of science education in Uganda specifically, we kindly request you to consider his application for authorization to conduct research in Uganda and provide him with necessary support he may require to successfully undertake this study.

Your kind cooperation is highly appreciated.

Yours sincerely,

Digitally signed by UR (Rukara, Directorate of Research& Innovation)
Date: 2020.10.26
Time: 11:31:16 + 2'00

Assoc. Prof. Eugene Ndabaga
Director of Research and Innovation
University of Rwanda- College of Education
E-mail: ndabagav@yahoo.ie
Mobile: **+250788308862**
Cc:

- The Principal, UR-CE
- Director, ACEITLMS, UR-CE
