# Supplementary material for: Dataset on the relationship between students’ attitude towards, and performance in mathematics word problems, mediated by active learning heuristic problem-solving approach
Source: Data Brief. 2023 Mar 14;48:109055. doi: 10.1016/j.dib.2023.109055 (PMC10051018; doi:10.1016/j.dib.2023.109055)
Supplement: Supplementary file 1 [file mmc1.zip › Supplementary material for DIB/Sample Linear Programming Word Problems.pdf]

## Sample Linear Programming Word Problems

**Q1.** A factory makes two kinds of bottle tops "Coca-cola" tops and "Pepsi cola tops". The same equipment can be used to make either. In making Coca-cola tops, one man can supervise 10machines and this batch will give a profit of pounds sterling (£) 50 per week. Pepsi cola tops yield a profit of (£) 250 a week, using 25 machines and 8 men. There are 200 machines and 40 men available. By taking $x$ batches of Coca-cola tops and $y$ batches of Pepsi cola tops; write down inequalities for the:

(i) number of machines used (ii) number of men employed (iii) expression for profit, P.

Use these inequalities to draw a suitable graph showing the region which satisfies them. From your graph, determine the numbers of Coca-cola tops and Pepsi cola tops which should be made to obtain the maximum profit. Hence find the maximum profit.

**Q2.** A wildlife club in a certain school wishes to go for an excursion to a national park. The club has hired a mini-bus and a bus to take students. Each trip for the bus is Shs.50.000 and that of a mini-bus is Shs.30.000. The bus has a capacity of 54 students and the minibus, 18 students. The maximum number of students allowed to go for the excursion is 216. The number of trips the bus makes do not have to exceed those made by the mini-bus. The club has mobilized as much as Shs.300,000 for transportation of the students. If $x$ and $y$ represent the number of trips made by the bus and mini-bus respectively,

(a) write down five inequalities representing the above information.
(b) plot these inequalities on the same axes.
(c) by shading, unwanted regions show the region satisfying the above inequalities.
(d) list the possible number of trips each vehicle can make.
(e) state the greatest number of students who went for the excursion.

**Q3.** A private car park is designed in such a way that it can accommodate $x$ pickups and $y$ minibuses at any given time. Each pickup is allowed 15m$^2$ of space and each miminibus5m$^2$ of space. There is only 400m$^2$ of space available for parking. Not more than 35 vehicles are allowed in the park at a time. Both types of vehicles are allowed in the park. But at most 10mini-buses are allowed at that time.

(a) (i) Write down all the inequalities to represent the above information.
   (ii) On the same axes plot the graphs to represent the above inequalities in (i) hence shade out unwanted regions.

(b) If the parking charges for pick up are Shs.500 and that for a minibus is Shs.800 per day, find how many vehicles of each type should be parked to obtain maximum income, hence find the daily maximum income.

**Q4.** A farmer plans to plant an 18hectare field with carrots and potatoes. The farmer's estimates for the project are shown in the table below.

| | **Carrots** | **Potatoes** |
|---|---|---|
| Harvesting cost @ ha | Shs.95, 000 | Shs.60,000 |
| Number of working hours | 12days | 4days |
| Expected profit per ha | Shs.228, 000 | Shs.157.000 |

The farmer has shs.1,140,000 only to invest in the project. The total number of working days is 120. By letting $x$ represent the number of hectares to be planted with carrots and $y$ the number of hectares to be planted with potatoes

(a) write down inequalities for the (i) cost of the project (ii) working days (iii) number of hectares used in the project (iv) the possibility that the field will at least be used for planting either carrots or potatoes.
(b) Write down an expression for the profit **P** in terms of ***x*** and ***y***
(c) (i) On the same axes, plot graphs of the inequalities in (a) and (b) above by shading out the unwanted regions.
(ii) Use your graph to determine how the farmer should use the field to maximize profit. Hence, find the farmer's maximum profit.

**Q5.** At a graduation party, the guests are served beer and soda. At least twice as many crates of beer as crates of soda are needed. A crate of beer contains 25 bottles and a crate of soda contains 24 bottles. More than 200 bottles of beer and soda are needed. A maximum of Shs.500, 000 may be spent on beer and soda. Assume a crate of beer costs shs.40, 000 and that of soda costcost.15, 000.
(a) (i) form inequalities to represent the above information.
(ii) represent the above inequalities on the same axes.
(iii) by shading the unwanted regions, represent the region satisfying the inequalities in (a) (i) above.
(b) From your graph, find the number of crates of beer and soda that should be bought if the cost is to be as low as possible. Find the amount that was paid for those crates of beer and soda.

**Q6.** A bicycle factory assembles two types of bicycles, Road master and Hero on different assembly lines. An assembly line for the road masters occupies an area of $60m^2$ of the floor space. The floor space available for all the assembly lines is $420m^2$. The assembly line of the road master needs 10 men to operate it and that of the hero needs 16 men to operate it. The assembly lines need a maximum of 120 men to operate them.
(a) If ***x*** and ***y*** represent the number of assembly lines for road master and hero respectively.
(i) form four inequalities to represent the given information.
(ii) draw graphs on the same axes to represent the inequalities in (a) (i) above. Shade the unwanted regions.

**Q7.** A private car park is designed in such a way that it can accommodate ***x*** pickups and ***y*** minibuses at any given time. Each pickup is allowed $15m^2$ of space and each miminibus$5m^2$ of space. There is only $400m^2$ of space available for parking. Not more than 35 vehicles are allowed in the park at a time. Both types of vehicles are allowed in the park. But at most 10mini-buses are allowed at a time.
(a) (i) Write down all the inequalities to represent the above information.
(ii) On the same axes plot the graphs to represent the above inequalities in (i) hence shade out unwanted regions.
(b) If the parking charges for pick up are Shs.500 and that for a minibus is Shs.800 per day, find how many vehicles of each type should be parked to obtain maximum income, hence find the daily maximum income.
