# Supplementary material for: Dataset on the relationship between students’ attitude towards, and performance in mathematics word problems, mediated by active learning heuristic problem-solving approach
Source: Data Brief. 2023 Mar 14;48:109055. doi: 10.1016/j.dib.2023.109055 (PMC10051018; doi:10.1016/j.dib.2023.109055)
Supplement: Supplementary file 1 [file mmc1.zip › Supplementary material for DIB/TLIPAT Post.pdf]

LINEAR PROGRAMMING ACHIEVEMENT TEST (POST-TEST)

Instructions:
Answer ALL the questions
Time allowed: 1 Hour, 20 minutes

Question 1

1(a) Represent the solution set of the inequality $x^2 - 4 \leq 0$ on the number line.

(b) Determine the coordinates of the point of intersection of:

(i) $3x - 4y - 12 = 0$ and the line $x = 0$

(ii) (ii) $y = 2x + 3$ *and* $3y - x - 4 = 0$

Question 2

A Geography club in a certain school wishes to go for the field work excursion to a national park. The club is to hire a **mini-bus** and a **bus** to take students. Each trip for the bus will cost Shs.**500,000** and that of a mini-bus will cost Shs.**300,000**. Due to Covid-19 pandemic, the bus will transport **36** students and the mini–bus, **9** students. The maximum number of students allowed to go for the excursion is **216**. The number of trips the bus makes do not exceed those made by the mini-bus. The club has mobilized Shs.**3,000,000** for the transportation of students. If ***x*** and ***y*** represent the number of trips made by the **bus** and **mini-bus** respectively,

(a) Write all the inequalities representing the above information.

(b) Plot the above inequalities in (a) above on the same coordinate axes.

(c) By shading the unwanted regions, write down the number of trips of mini-buses and buses needed to minimize the transport costs of the fieldwork excursion.

Question 3

Use the figure below to answer the following questions:

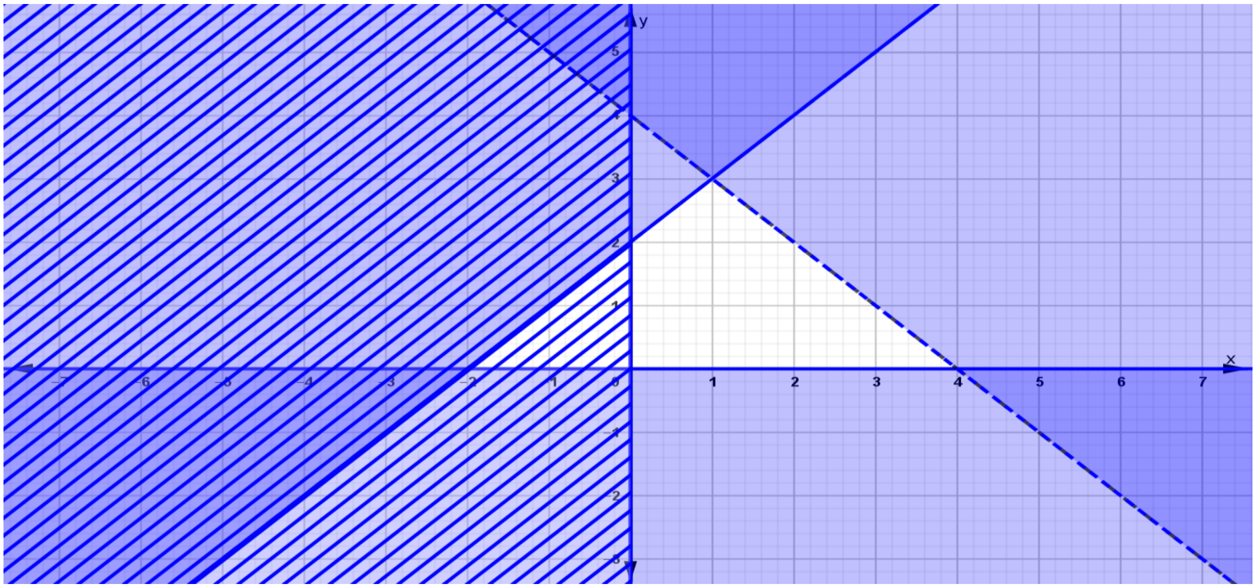

(a) Write down all the inequalities satisfying the unshaded (feasible) region.

(b) Write down the integral coordinates of the points which lie in the feasible region.

(c) Determine the maximum value of $3x - y$.

The end
